# Divergent effects of adrenaline in human induced pluripotent stem cell-derived cardiomyocytes obtained from hypertrophic cardiomyopathy

**DOI:** 10.1242/dmm.032896

**Published:** 2018-02-01

**Authors:** Chandra Prajapati, Marisa Ojala, Katriina Aalto-Setälä

**Affiliations:** 1 BioMediTech, University of Tampere, 33014 Tampere, Finland; 2 Faculty of Medicine and Life Science, University of Tampere, 33014 Tampere, Finland; 3 Heart Hospital, Tampere University Hospital, 33521 Tampere, Finland

**Keywords:** HCM, Arrhythmia, hiPSC-CMs, Adrenaline, Bisoprolol

## Abstract

Hypertrophic cardiomyopathy (HCM) is a common inherited cardiac disease that affects the heart muscle with diverse clinical outcomes. HCM can cause sudden cardiac death (SCD) during or immediately after mild to rigorous physical activity in young patients. However, the mechanism causing SCD as a result of exercise remains unknown, but exercise-induced ventricular arrhythmias are thought to be responsible for this fatal consequence. To understand the disease mechanism behind HCM in a better way, we generated patient-specific induced pluripotent stem cell-derived cardiomyocytes (hiPSC-CMs) from HCM patients carrying either the *MYBPC3-Gln1061X* or *TPM1-Asp175Asn* mutation. We extensively investigated the effects of low to high concentrations of adrenaline on action potential characteristics, and the occurrence of arrhythmias in the presence of various concentrations of adrenaline and in wash-out condition. We classified and quantified different types of arrhythmias observed in hiPSC-CMs, and found that the occurrence of arrhythmias was dependent on concentrations of adrenaline and positions of mutations in genes causing HCM. In addition, we observed ventricular tachycardia types of arrhythmias in hiPSC-CMs carrying the *TPM1-Asp175Asn* mutation. We additionally examined the antiarrhythmic potency of bisoprolol in HCM-specific hiPSC-CMs. However, bisoprolol could not reduce the occurrence of arrhythmias during administration or during the wash-out condition of adrenaline in HCM-specific hiPSC-CMs. Our study demonstrates hiPSC-CMs as a promising tool for studying HCM. The experimental design used in this study could be suitable and beneficial for studying other components and drugs related to cardiac disease in general.

## Abstract (precis)

**Summary:** Different concentrations of adrenaline have divergent effects during and immediately after administration in human induced pluripotent stem cell-derived cardiomyocytes (hiPSC-CMs) obtained from hypertrophic cardiomyopathy (HCM) patients. Bisoprolol could not reduce the arrhythmias in HCM-specific hiPSC-CMs.

## INTRODUCTION

Hypertrophic cardiomyopathy (HCM) is a common genetic cardiac disease found worldwide, irrespective of age, sex or ethnic group. HCM is characterized by unexplained left ventricular hypertrophy, mostly in the interventricular septum, but it can also affect other areas of the left ventricle (Maron and Maron, 2013). Histologically, HCM is characterized by myocardial disarray and fibrosis. More than 1400 distinct mutations in >11 genes encoding proteins of cardiac sarcomeres have been associated with HCM, most of which are unique to individual families (Maron and Maron, 2013). Two founder mutations in alpha-tropomyosin (*TPM1-Asp175Asn*, 6.5%) and in myosin-binding protein C (*MYBPC3-Gln1061X*, 11.4%) genes together account for ∼18% of HCM in Finland (Jääskeläinen et al., 2013). HCM patients often have ventricular arrhythmias such as non-sustained ventricular tachycardia (NSVT) (Monserrat et al., 2003) and supraventricular arrhythmias such as atrial fibrillation (Adabag et al., 2005). In worst cases, the first manifestation of HCM could be sudden cardiac death (SCD), usually caused by ventricular tachyarrhythmias (Maron and Maron, 2014). It is known that HCM undergoes remodeling of different ion channels, but how these adaptations endorse repolarization abnormalities and engender lethal arrhythmias is not yet understood (Tomaselli and Marbán, 1999). The reduced number of β-receptor binding sites (Choudhury et al., 1996) and the blunt β-adrenergic signaling pathway have been identified, but interestingly the plasma catecholamine concentration remains unaltered in HCM patients (Schumacher et al., 1995). An earlier study showed that plasma adrenaline does not significantly differ in resting conditions and during exercise between control and HCM groups (Omodani et al., 1998). However, SCD occurs during or immediately after moderate to severe physical activity in HCM patients (van Rijsingen et al., 2011). Although exercise-induced arrhythmias have been associated with an increased risk of SCD, the precise mechanism for SCD has not yet been identified (Gimeno et al., 2009). HCM patients are recommended not to participate in competitive exercise because of impaired hemodynamics and tolerance during exercise (Elliott et al., 2014). Clinically, study of exercise-induced arrhythmias in HCM is challenging. In an early study, eight of 15 HCM patients stopped exercise because of shortness of breath or leg fatigue (Omodani et al., 1998). Currently, there is no disease-specific pharmacological treatment available for HCM patients (Spoladore et al., 2012); an implantable cardioverter-defibrillator (ICD) is the only effective available tool for prevention of SCD (Maron et al., 2000). Therefore, a better understanding of the triggers for lethal ventricular arrhythmias in HCM is necessary.

Prior studies of cardiovascular diseases in animal models have some fundamental problems, such as differences in cardiac physiology, drug responses (O'Hara and Rudy, 2012), and the expression of contractile proteins. Therefore, it is complicated to extrapolate the physiological and pharmacological results from animals to humans (Jung and Bernstein, 2014). In addition, cardiac biopsies from humans are limited and typically obtained from the end stages of cardiac diseases (Barajas-Martínez et al., 2013); hence, it is not possible to understand the mechanisms leading to cardiac diseases. These impedances of *in vitro* cardiac disease modeling are mostly overcome by the groundbreaking discovery of reprogramming adult somatic cells into induced pluripotent stem cells (iPSCs) (Takahashi et al., 2007), which can be differentiated into any cell type of the human organism, such as human iPSC-derived cardiomyocytes (hiPSC-CMs) (Kujala et al., 2012; Ojala et al., 2016). In addition, hiPSC-CMs offer a robust platform for *in vitro* studies of genetic cardiac disorders. We have previously shown that HCM-specific hiPSC-CMs carrying *TPM1-Asp175Asn* or *MYBPC3-Gln1061X* mutations showed differences in cell morphology, calcium handling properties and electrophysiological properties, not only from the control but also between these two mutations (Ojala et al., 2016). This study is a follow-up study with comprehensive analysis of the effects of different concentrations of adrenaline on action potential (AP) characteristics and the occurrence of arrhythmias in HCM-specific hiPSC-CMs during and immediately after adrenaline administration. We concentrated on the use of adrenaline because it is a natural physiological agent and plasma adrenaline increases during exercise (Omodani et al., 1998; Warren et al., 1984). Furthermore, we evaluated the beat rate and repolarization variabilities of these hiPSC-CMs to identify the potential link with the occurrence of arrhythmias. Finally, we also examined the efficacy of the β-blocker bisoprolol in subsiding arrhythmias in HCM-specific hiPSC-CMs.

## RESULTS

### Electrophysiological categorization of hiPSC-CMs

In this study, hiPSC-CMs were categorized as ventricular-like [action potential duration (APD) at 90%/50% repolarization (APD90/APD50)<1.3] and atrial-like (APD90/APD50>1.35) mainly based on the triangularity of the AP profile.

#### Atrial-like hiPSC-CMs

The minority (∼20%) of the hiPSC-CMs exhibited the atrial type of AP profile in hiPSC-CMs derived from control hiPSCs (WT-CMs), hiPSC-CMs carrying the *TPM1-Asp175Asn* mutation (HCMT-CMs) and hiPSC-CMs carrying the *MYBPC3-Gln1061X* mutation (HCMM-CMs). Only baseline characteristics were evaluated from atrial-like hiPSC-CMs and none of the AP characteristics of atrial cells were significantly different among WT-CMs (*n*=21), HCMT-CMs (*n*=30) and HCMM-CMs (*n*=34) (Table S1).

#### Ventricular-like hiPSC-CMs

The majority (∼80%) of the hiPSC-CMs exhibited the ventricular-like hiPSC-CMs in all groups. The baseline characteristics of ventricular-like hiPSC-CMs have been presented in our previous study (Ojala et al., 2016). In this study, adrenaline and bisoprolol testing was performed only in ventricular-like hiPSC-CMs.

### Voltage-gated ionic currents in hiPSC-CMs

**Fig. 1.**
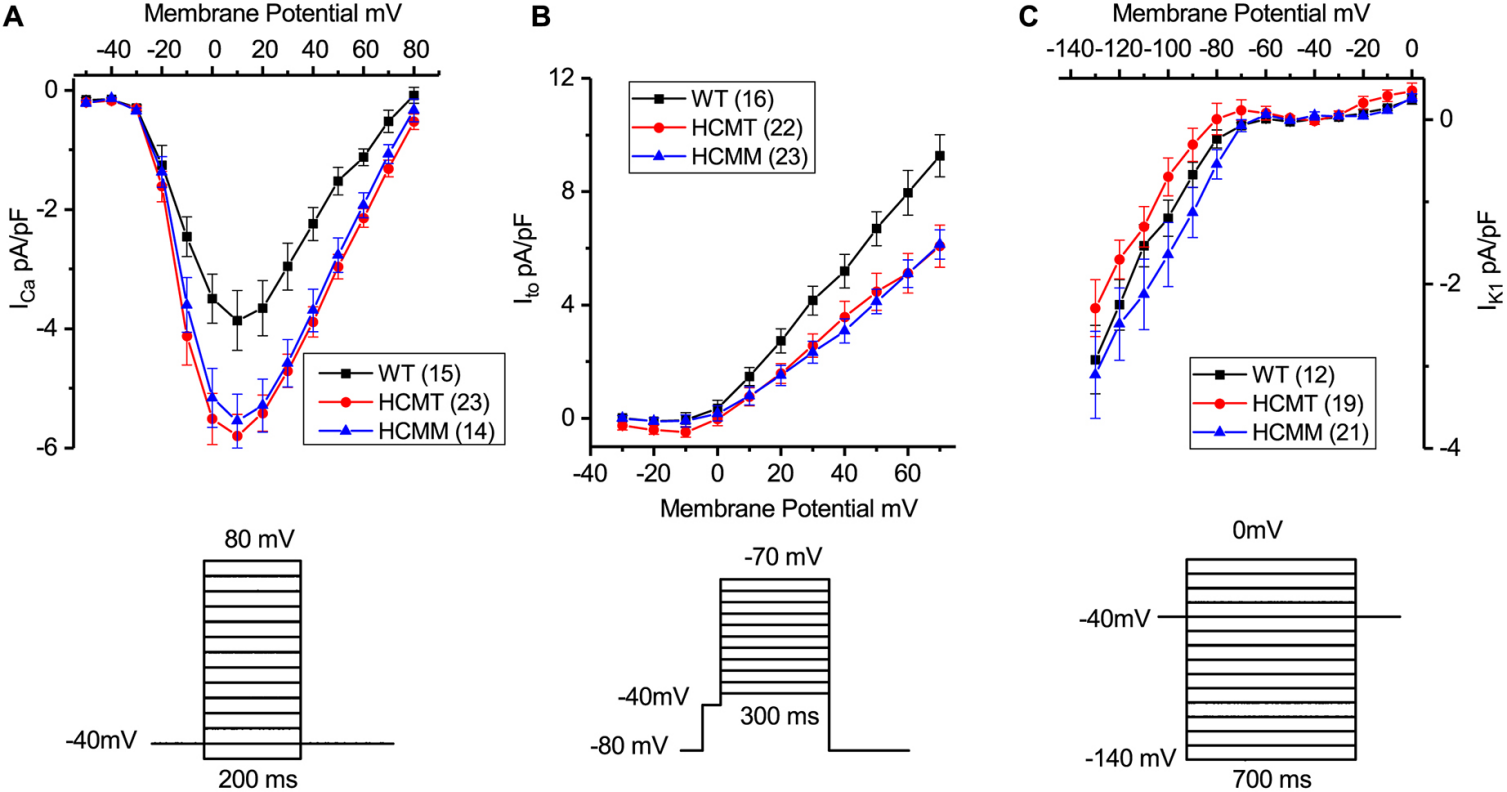
**Voltage-gated ionic currents in ventricle-like WT-CMs, HCMT-CMs and HCMM-CMs.** (A) Current-voltage (I-V) relationship of L-type calcium current (I_Ca_) (above) and voltage clamp protocol used (below). *P*<0.05 from −10 mV to 60 mV (WT-CMs versus HCMT-CMs, one-way ANOVA, post hoc Tukey test). *P*<0.05 from 10 mV to 60 mV (WT-CMs versus HCMM-CMs, one-way ANOVA, post hoc Tukey test). (B) I-V relationship of transient outward potassium current (I_to_) (above) and voltage clamp protocol used (below). *P*<0.05 from 30 mV to 70 mV (WT-CMs versus HCMT-CMs and WT-CMs versus HCMM-CMs; one-way ANOVA, post hoc Tukey test). (C) I-V relationship of inward rectifier outward current (I_K1_) (above) and voltage clamp protocol used (below) (*ns* at all test potentials, WT-CMs versus HCMT-CMs and WT-CMs versus HCMM-CMs; one-way ANOVA, post hoc Tukey test). Data are mean±s.e.m. Values inside parentheses represent the number of CMs used.

Previous studies have shown that either gain or loss of function of different voltage-gated ion channels alter the AP profile, and thus facilitate the occurrence of arrhythmias in HCM (Coppini et al., 2013; Gomez et al., 1997). To understand the remodeling of ion channels in HCM, calcium current (I_Ca_), transient outward potassium current (I_to_) and inward rectifier potassium current (I_K1_) were measured in hiPSC-CMs (Fig. 1). The I_Ca_ current densities were significantly higher in HCMT-CMs (*n*=23) and HCMM-CMs (*n*=14) than in WT-CMs (*n*=15) (*P*<0.05, WT versus HCMT, from −10 mV to 70 mV, and WT versus HCMM, from 10 mV to 60 mV; one-way ANOVA, post hoc Tukey test). However, no statistical differences in I_Ca_ current densities were found between HCMT-CMs and HCMM-CMs at any potential (Fig. 1A). By contrast, I_to_ current densities were significantly lower in both HCMT-CMs (*n*=22) and HCMM-CMs (*n*=23) compared to WT-CMs (*n*=16) from 20 mV to 70 mV (*P*<0.05, one-way ANOVA, post hoc Tukey test; Fig. 1B). No significant differences in I_to_ current densities were found between HCMT-CMs and HCMM-CMs at any potential. The I_K1_ current densities were not significantly different at all potentials tested among groups [nonsignificant (*ns*), WT-CMs, *n*=12; HCMT-CMs, *n*=19; HCMM-CMs, *n*=21; one-way ANOVA, post hoc Tukey test; Fig. 1C].

### hiPSC-CMs exhibit various types of arrhythmias

**Fig. 2.**
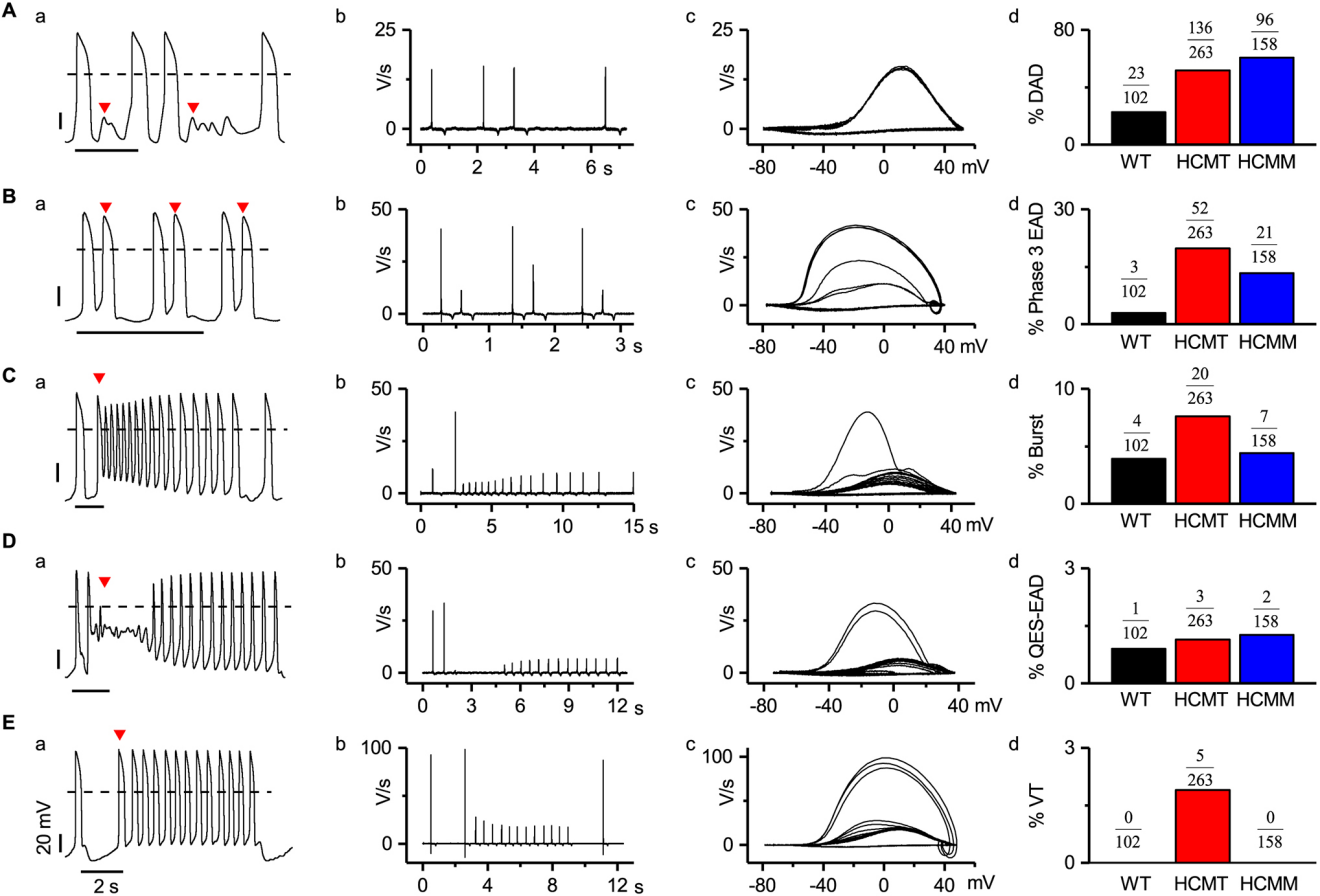
**Classification of arrhythmias recorded in ventricle-like hiPSC-CMs.** (A**)** Representative AP recording with DADs (Aa), its first derivative (Ab), its phase plot (Ac) and percentage of cells exhibiting DADs (Ad). (B**)** Representative AP recording with phase-3 EAD (Ba), its first derivative (Bb), its phase plot (Bc) and percentage of cells exhibiting phase-3 EAD (Bd). (C) Representative AP recording with burst EAD (Ca), its first derivative (Cb), its phase plot (Cc) and its occurrence, calculated as number of bursts/total number of cells (Cd). (D**)** Representative AP recording with QES-EAD (Da), its first derivative (Db), its phase plot (Dc) and its occurrence, calculated as number of QES-EADs/total number of cells (Dd). (E) Representative AP recording with VT-EAD (Ea), its first derivative (Eb), its phase plot (Ec) and percentage of cells exhibiting VT (Ed). Arrowheads indicate the corresponding arrhythmias in APs. Dashed line represents 0 mV. (For more detail, see Fig. S3.)

**Fig. 3.**
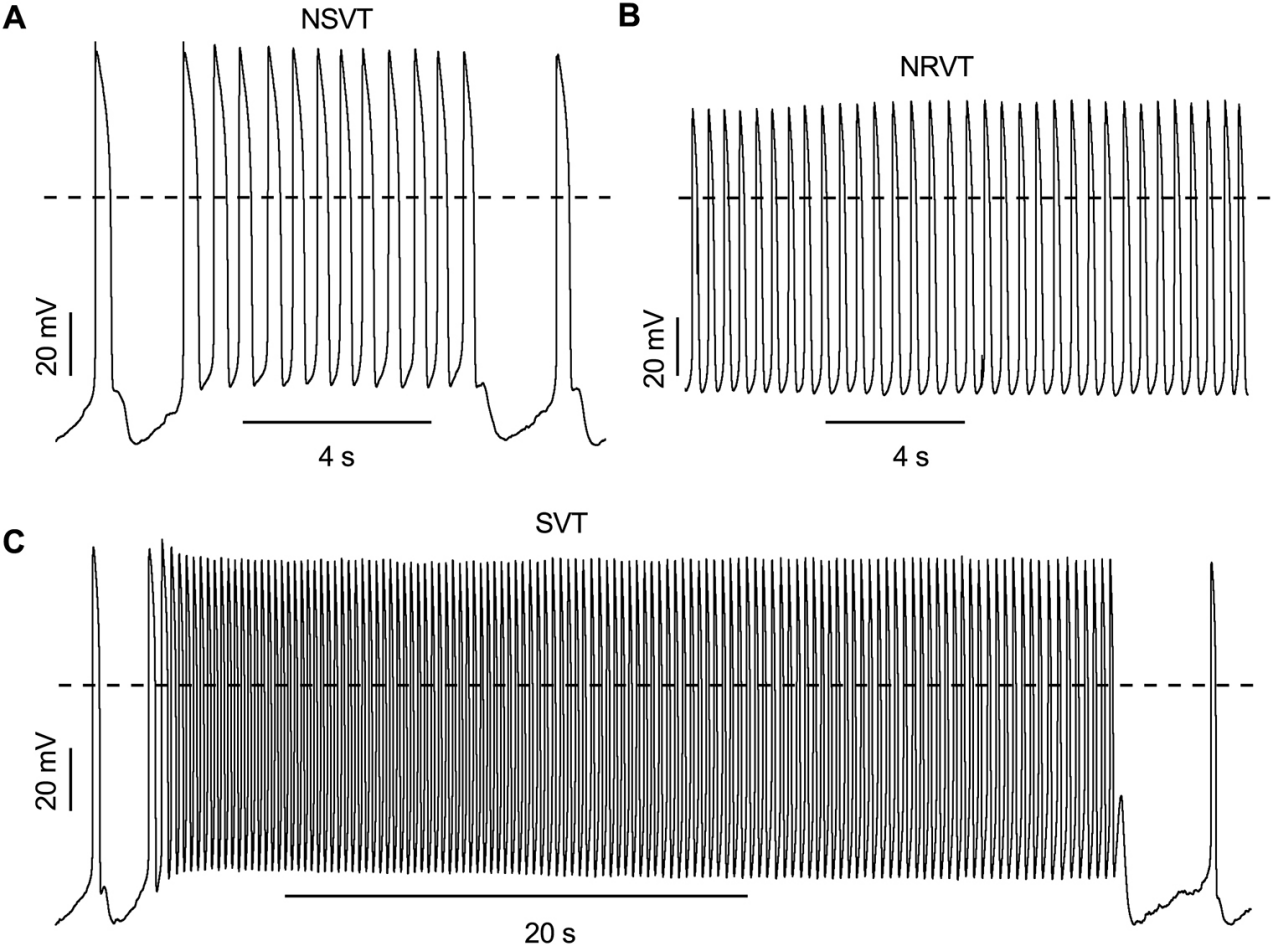
**Representative AP profile of VT arrhythmias recorded in HCMT-CMs.** (A) AP with non-sustained ventricular tachycardia (NSVT, duration <30 s) (B) non-recovered ventricular tachycardia (NRVT) and (C) sustained ventricular tachycardia (SVT, >30 s). Dashed line represents 0 mV.

Life-threatening arrhythmias and exercise-induced arrhythmias primarily commence in the ventricle (Monserrat et al., 2003; Gimeno et al., 2009). Thus, we especially focused on ventricular-like hiPSC-CMs to understand these abnormalities. Patch-clamp recordings of self-beating hiPSC-CMs demonstrated various types of arrhythmias (Fig. 2; Fig. S1). Therefore, we categorized and quantified these arrhythmias separately. The arrhythmias were present either exclusively or in the presence of another type of arrhythmia. The most common type of arrhythmia recorded from hiPSC-CMs was delayed afterdepolarization (DAD). DAD was defined as the presence of low-amplitude abnormal depolarization after successive APs that can cause cessation of next spontaneous APs (Fig. 2Aa; Fig. S1A). Both HCMT-CMs (52%, 136/263) and HCMM-CMs (61%, 96/158) exhibited higher occurrence of DADs compared to WT-CMs (23%, 23/102) at baseline (Fig. 2Ad). DADs were also recorded from the atrial hiPSC-CMs (Fig. S2A) in WT-CMs (5%, 1/21), HCMT-CMs (43%, 13/30) and HCMM-CMs (18%, 6/34). The second type of arrhythmia recorded from hiPSC-CMs was phase 3 early afterdepolarization (EAD). Phase 3 was defined as the initiation of the next AP during the third phase of repolarization, i.e. phase 3 in preceding APs (Fig. 2Ba; Fig. S1B). EAD-induced triggered activity in hiPSC-CMs displayed take-off potentials ranging between −55 mV and −65 mV. When the AP comprising phase 3 EAD was closely analyzed, two distinctive patterns of upstroke velocity were observed. Either the upstroke velocity of AP constituting phase 3 EAD increased compared to its previous AP, or it remained the same (Fig. 2Bb,c). In the baseline condition, the percentage of cells exhibiting phase 3 EAD was higher in both HCMT-CMs (20%, 52/263) and HCMM-CMs (13%, 21/158) than in WT-CMs (3%, 3/102) (Fig. 2Bd). Phase 3 EADs were also recorded from the atrial hiPSC-CMs (Fig. S2B) from WT-CMs (5%, 1/21), HCMT-CMs (13%, 4/30) and HCMM-CMs (3%, 1/34). The third type of arrhythmia exhibited in hiPSC-CMs was burst EAD. This type of EAD was defined as the sudden increase in beating rate triggered by phase 3 EAD (Fig. 2Ca; Fig. S1C). The main characteristics of burst EAD are a faster beat rate and continuous change (either increasing or decreasing) in action potential amplitude (APA). Similar to phase 3 EAD, another notable characteristic observed in some of the burst was an increase in the upstroke velocity in AP comprising burst EAD (Fig. 2Cb,c). The length and beat rate of the burst EAD observed in hiPSC-CMs ranged from 0.3 s to 27 s and from 50 beats per minute (BPM) to 260 BPM, respectively. Similarly, the take-off potential for burst in our study ranged from −70 mV to −30 mV. Furthermore, the occurrence of burst was two times higher in HCMT-CMs (8%, 20/263) than in HCMM-CMs (4%, 7/158) and WT-CMs (4%, 4/102) in baseline conditions (Fig. 2Cd). Similarly, bursts were also observed in atrial-like hiPSC-CMs (Fig. S2C) from HCMT-CMs (3%, 1/30) and HCMM-CMs (3%, 1/34), but not in those from WT-CMs (0%, 0/21). The fourth type of arrhythmia recorded from hiPSC-CMs was quasi-equilibrium state (QES) EAD (QES-EAD), which was defined as a sudden decrease in APA leading to temporary non-beating membrane potential or oscillation and self-recovery to spontaneous APs (Fig. 2Da; Fig. S1D). As in phase 3 EAD and burst EAD, an increase in upstroke velocity in AP comprising QES-EAD was observed in some cases (Fig. 2Db,c). The take-off potential and duration of QES-EAD recorded in our study varied from −55 mV to −6.5 mV and from 1.4 s to 22.3 s, respectively. At baseline conditions, QES-EADs were observed at low incidence in WT-CMs (1%, 1/102), HCMT-CMs (1%, 3/263) and HCMM-CMs (1%, 2/158) (Fig. 2Dd). However, QES-EADs were not observed in atrial-like hiPSC-CMs. The fifth type of arrhythmia observed in hiPSC-CMs was ventricular tachycardia (VT), which was defined as phase 3 EAD mediating triggered activity followed by an increased beating rate (Fig. 2Ea; Fig. 3). The main characteristics of VT-EAD were a steady APD and maximum diastolic potential (MDP) during triggered activity. Our results showed that the beat rate and MDP of VT ranged from 70 BPM to 254 BPM and from −69 mV to −45 mV, respectively. Furthermore, another distinguished feature of VT was the possibility of progressively extended triggered activity. Therefore, VTs were subdivided by duration as non-sustained (NSVT, duration <30 s, self-terminated; Fig. 3A), sustained (SVT, duration>30 s, self-terminated; Fig. 3C) and non-recovered (NRVT, not recovered; Fig. 3B). Unlike other types of EAD, an alternation in upstroke velocity constituting VT was not observed. The first four types of arrhythmia (DAD, phase 3 EAD, burst and QES-EAD) were observed in WT-CMs, HCMT-CMs and HCMM-CMs. Interestingly, VTs were only found in HCMT-CMs. To confirm that VTs were exclusively present in HCMT-CMs, we decided to include the clonal line of UTA.02912.HCMT with the *TPM1-Asp175Asn* mutation called UTA.02913.HCMT (Table S2, Fig. S3). In current clamp recording, we were also able to record VTs in hiPSC-CMs derived from the UTA.02913.HCMT line and therefore confirmed that VT was present in HCMT-CMs.

### Baseline variabilities in hiPSC-CMs

**Fig. 4.**
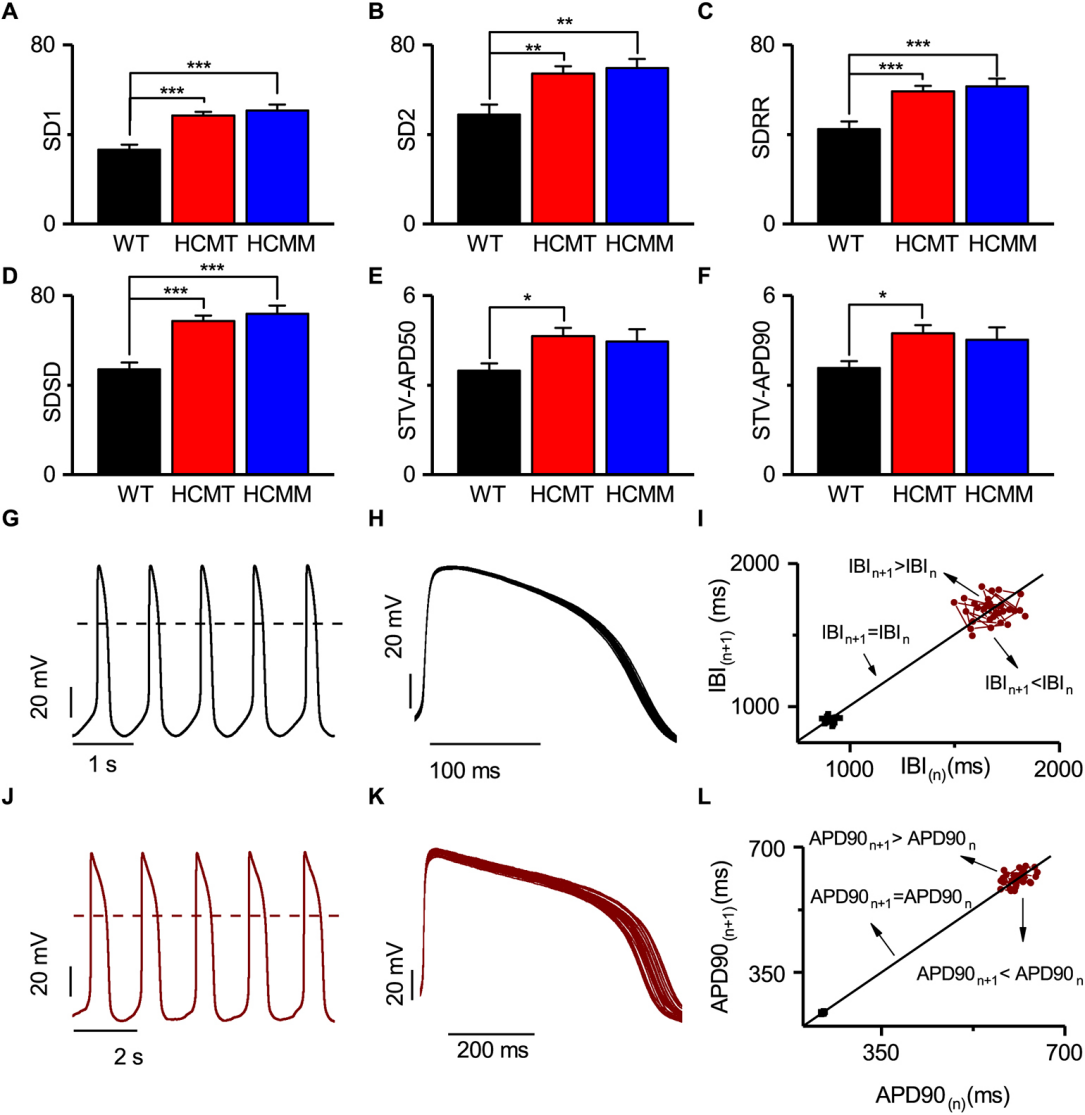
**Summary of variabilities in hiPSC-CMs.** (A-F) Comparison of SD1, SD2, SDRR, SDSD, STV-APD50 and STV-APD90 in WT-CMs (*n*=63), HCMT-CMs (*n*=154) and HCMM-CMs (*n*=79). Data are mean±s.e.m. *P<0.05, **P<0.005 and ***P<0.0001; one-way ANOVA, post hoc Tukey test). (G-L) Representative AP traces (G,J), with superimposed 30 APs (H,K) and Poincaré plots of these APs from hiPSCs exhibiting low (black circles) and high (maroon circles) variabilities (I,L). Dashed line represents 0 mV. (For more detail, see Figs S6-S9.)

Clinically, it is well known that heart rate variabilities and/or ventricular repolarization variabilities differ between control individuals and diseased patients, and are associated with arrhythmias present in the patients (Counihan et al., 1993). In addition, an earlier study showed that hiPSC-CMs exhibited beat rate variability (Mandel et al., 2012). Thus, the beat rate variabilities [standard deviation of instantaneous variability (SD1), standard deviation of long-term variability (SD2), standard deviation of differences between adjacent RR intervals (SDSD) and standard deviation of all RR intervals (SDRR)] and repolarization variabilities [beat-to-beat variability at 50% or 90% repolarization (STV-APD50 or STV-APD90)] were measured to understand whether these variabilities differ in WT-CMs, HCMT-CMs and HCMM-CMs. Thus, recordings of hiPSC-CMs with ≥30 consecutive APs without the presence of any type of arrhythmia were selected from WT-CMs (*n*=79), HCMT-CMs (*n*=174) and HCMM-CMs (*n*=118). First, the three-dimensional plots among SD1, beat rate and APD90, and STV-APD90, beat rate and APD90, were plotted (Fig. S4). After that, correlation tests were performed among beat rate, beat rate and repolarization variabilities, and APDs. The results showed a negative correlation between beat rate and beat rate variabilities in all groups, i.e. the higher the beat rate, the lower the beat rate variability (Fig. S5A-F). By contrast, we found a positive correlation between APDs and APD variability in all groups, i.e. the longer the APD, the higher the STV (Fig. S5G-L). On the other hand, the results of correlation tests between beat rate variabilities and repolarization variabilities showed a positive relationship between SD1 and APD90 in HCMT-CMs and HCMM-CMs (*P*<0.05, Pearson's correlation) but not in WT-CMs (Fig. S6). Therefore, to minimize the effects of beat rate and APDs in their respective variabilities to compare among groups, variabilities data from CMs were again filtered. The newly selected CMs had beat rates ranging from 30 BPM to 60 BPM, and also APDs of 200-600 ms. Consequently, the mean beat rates (WT-CMs 52.5±1.4 versus HCMT-CMs 52.1±1.0 versus HCMM-CMs 55.8±1.3), APD50 (WT-CMs 282.1±8.3 versus HCMT-CMs 283.3±6.7 versus HCMM-CMs 278.9±8.9) and APD90 (WT-CMs 333.6±9.0 versus HCMT-CMs 334.9±7.4 versus HCMM-CMs 328.2±9.6) were not significantly different among the groups (*ns*, one-way ANOVA, post hoc Tukey test; WT-CMs *n*=63, HCMT-CMs *n*=154 and HCMM-CMs *n*=119; Fig. S7). Thus, we compared variabilities among groups. Fig. 4 shows the variabilities parameters in WT-CMs, HCMT-CMs and HCMM-CMs. All the beat rate variabilities parameters, such as SD1, SD2, SDRR and SDSD, were significantly higher (*P*<0.05, one-way ANOVA, post hoc Tukey test) in HCMT-CMs and HCMM-CMs than in WT-CMs (Fig. 4A-F). These results imply that both HCMT-CMs and HCMM-CMs exhibit higher degrees of beat variability than WT-CMs. However, STV-APD90 and STV-APD50 were only significantly higher (*P*<0.05, one-way ANOVA, post hoc Tukey test) in HCMT-CMs compared to WT-CMs (Fig. 4E,F). The CMs exhibiting a higher degree of beat rate and repolarization variabilities were more scattered in Poincaré plot (Fig. 4I and Fig. 3L). In addition, beat rate variabilities and repolarization variabilities of atrial-like hiPSC-CMs from WT-CMs (*n*=21), HCMT-CMs (*n*=27) and HCMM-CMs (*n*=33) were measured, but no significant differences (*ns*, one-way ANOVA, post hoc Tukey test) in any parameters were found among groups (Table S3).

### Adrenaline testing in hiPSC-CMs

Adrenaline levels increase during exercise (Warren et al., 1984), and HCM patients and healthy individuals have similar plasma adrenaline levels during rest and exercise conditions (Omodani et al., 1998). However, HCM patients experience lethal arrhythmia during or immediately after exercise (Gimeno et al., 2009). To understand whether adrenaline causes any adverse effects, hiPSC-CMs were exposed to different concentrations of adrenaline, including 0.1 nM, 0.5 nM, 1 nM, 10 nM, 100 nM, 1 µM and 10 µM. The hiPSC-CMs were administrated only one concentration at a time to avoid the possible effect of desensitization of the receptor (Priori and Corr, 1990). The experimental protocol for the adrenaline testing is shown in Fig. S8.

#### Effects on AP characteristics during the administration of adrenaline

**Fig. 5.**
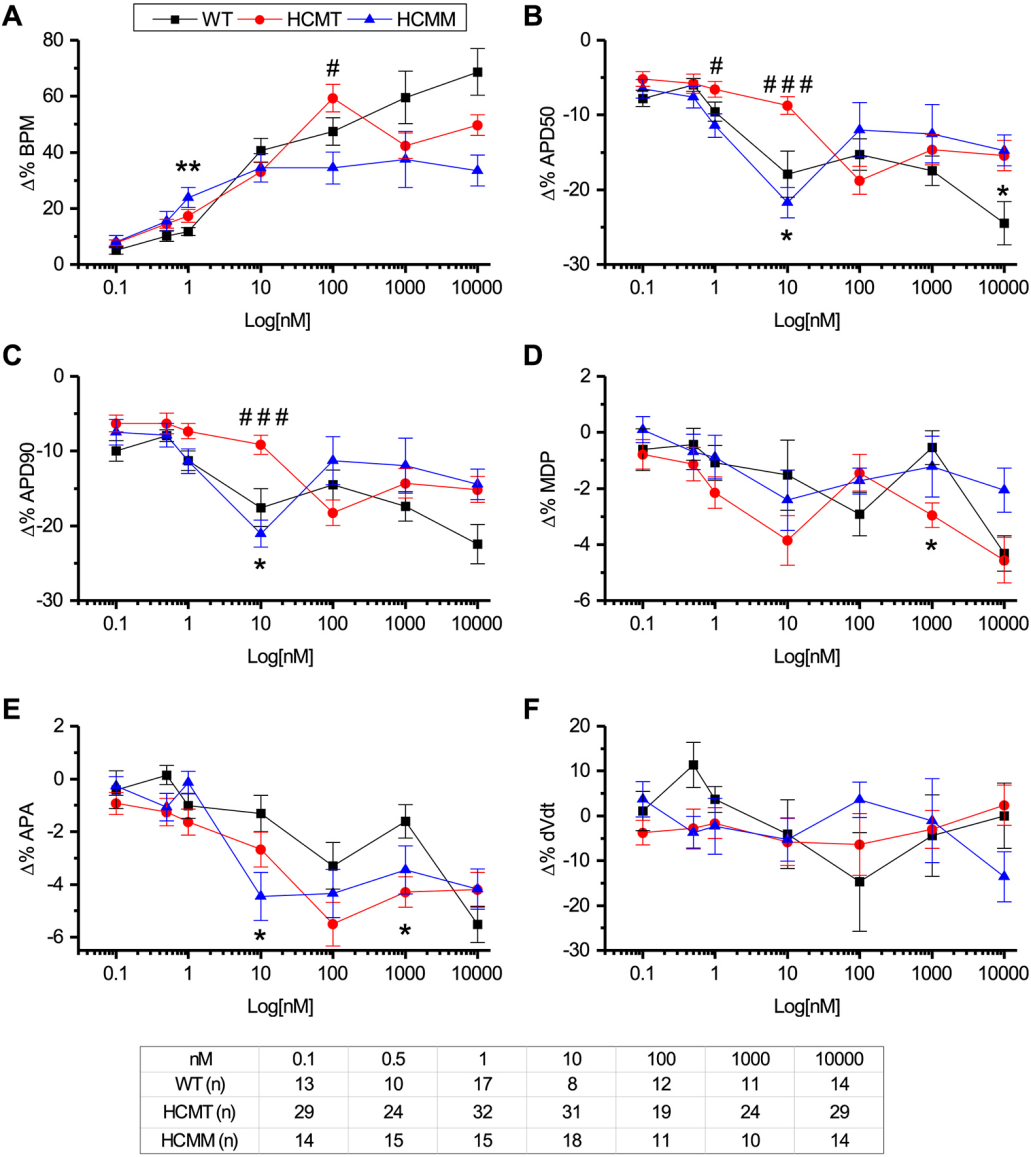
**Superimposed traces representing the effects of different concentrations of adrenaline on AP characteristics.** (A-F) Percentage change in BPM (A), APD50 (B), APD90, (C) MDP, (D) APA (E) and dV/dt (F), with respect to pre-adrenaline administration condition in WT-CMs, HCMT-CMs and HCMM-CMs. One concentration of adrenaline was tested at a time. Data are mean±s.e.m. *P<0.05, WT versus HCMT or HCMM; ^#^*P*<0.05, HCMT versus HCMM; one-way ANOVA, post hoc Tukey test. Table shows the number of cells used in each group at different adrenaline concentrations. (For more detail, see Tables S4-S7.)

The effects of adrenaline were calculated as the percentage change compared to pre-adrenaline administration. Fig. 5 summarizes the effects of various concentrations of adrenaline on AP characteristics in WT-CMs, HCMT-CMs and HCMM-CMs. The beat rates were significantly increased (*P*<0.05, paired *t*-test), but APD50 and APD90 were significantly decreased (*P*<0.05, paired *t*-test), from their pre-adrenaline values in a concentration-dependent manner in WT-CMs (Table S4), HCMT-CMs (Table S5) and HCMM-CMs (Table S6) (Fig. 5A-C). Interestingly, APD50 and APD90 were similarly reduced by adrenaline at all concentrations of adrenaline (*P*<0.0001, APD50 versus APD90, Pearson's correlation; data not shown). Notably, upstroke velocity (dV/dt) was not significantly changed by any concentration of adrenaline in WT-CMs, HCMT-CMs and HCMM-CMs (*P*<0.05, paired *t*-test; Tables S4-S6). The percentage changes in beat rate and APD90 were not always dependent on their pre-adrenaline values or on one another, as shown by correlation tests (Table S7).

#### Effects of adrenaline, during and immediately after administration, on the occurrence of arrhythmias

To study the effects of adrenaline on arrhythmia frequencies, we quantified those arrhythmias before exposure to adrenaline (baseline condition), during the administration of adrenaline, and immediately after washing off the adrenaline. Table S8 shows the DAD rate in three different conditions in WT-CMs, HCMT-CMs and HCMM-CMs. No significant differences in DAD rate when comparing before, during and immediately after the wash-out condition of different concentrations of adrenaline were observed in WT-CMs, HCMT-CMs and HCMM-CMs (*ns*, Friedman, post hoc Dunn test) at any concentration tested (Table S8). Similarly, phase 3 EAD rate at different concentrations were also quantified, but significant changes were not observed at any concentration in any group (*ns*, Friedman, post hoc Dunn test; Table S9). At higher concentrations of adrenaline (>1 nM), bursts were not observed in WT-CMs or HCMM-CM in the presence of adrenaline (Table S10). However, bursts were recorded at all concentrations of adrenaline tested both during administration and after washing out adrenaline in HCMT-CMs (Table S10). Similarly, QES-EADs were observed during the administration of adrenaline, except at the 100 nM concentration in HCMT-CMs (Table S11). By contrast, QES-EADs were observed in all three conditions at 100 nM adrenaline in WT-CMs (Table S11). Furthermore, when studying the occurrence of different types of VTs in HCMT-CMs, only NSVTs were observed at baseline conditions. However, transition from NSVT to sustained SVT and/or NRVT was observed during the administration and wash-out condition of adrenaline (Fig. 3; Table S12). NSVTs were observed during the wash-out conditions from the 100 nM and 1 µM concentrations of adrenaline (Table S12).

#### Effects on variabilities during the administration of adrenaline

**Fig. 6.**
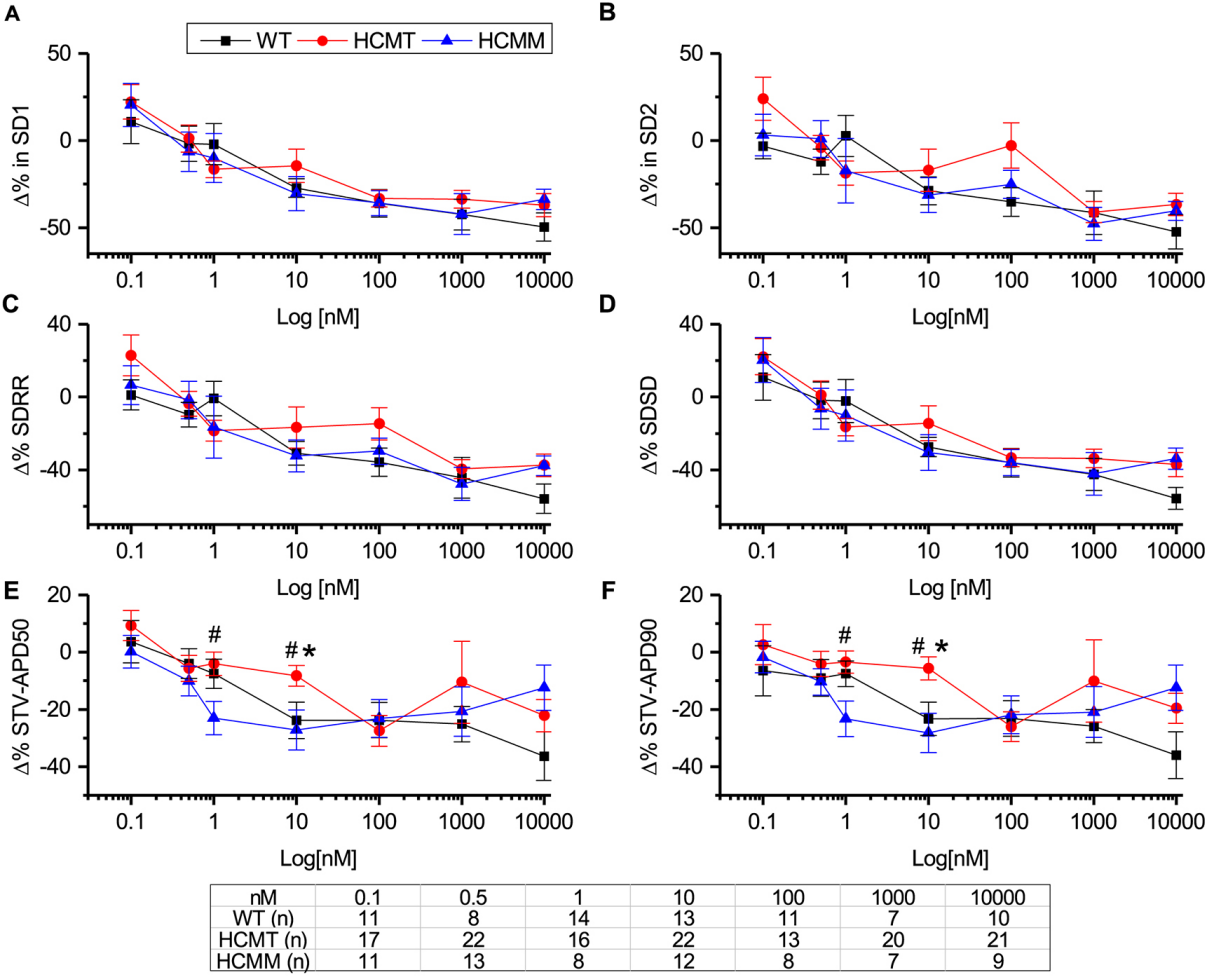
**Superimposed traces representing the effects of different concentrations of adrenaline on beat rate and repolarization variabilities.** (A-F) Percentage change in SD1 (A), SD2 (B), SDRR (C), SDSD (D), STV-APD50 (E) and STV-APD90 (F) with respect to pre-adrenaline administration condition in WT-CMs, HCMT-CMs and HCMM-CMs. Data are mean±s.e.m. *P<0.05, WT-CMs versus HCMT-CMs or HCMM-CMs; ^#^*P*<0.05, HCMT-CMs versus HCMM-CMs; one-way ANOVA, post hoc Tukey test. Table shows the number of cells used in each group at different adrenaline concentrations. (For more detail, see Tables S13-15.)

We also quantified the beat rate variabilities and repolarization variabilities in hiPSC-CMs after the administration of adrenaline. Similarly, as before, the percentage changes in variabilities were calculated with respect to pre-adrenaline administration. Fig. 6A-D and Fig. 6E,F summarize the changes in beat rate variabilities and repolarization variabilities, respectively, with various concentrations of adrenaline. There were no major significant differences in beat rate variabilities in WT-CMs, HCMT-CMs and HCMM-CMs at any adrenaline concentration (*ns*, one-way ANOVA, post hoc Tukey test). Only the percentage changes in both repolarization variabilities were significantly less at 1 nM and 10 nM of adrenaline in HCMT-CMs when compared to HCMM-CMs or WT-CMs (*P<0.05, WT versus HCMT at 10 nM adrenaline; ^#^*P*<0.05, HCMT versus HCMM at 1 nM adrenaline and 10 nM adrenaline; one-way ANOVA, post hoc Tukey test; Fig. 6E,F). Although beat rates were increased and APDs were decreased at increasing adrenaline concentrations, beat rate and repolarization variabilities were not decreased in a similar fashion in WT-CMs (Table S13), HCMT-CMs (Table S14) and HCMM-CMs (Table S15).

### Pharmacological treatment of HCM-specific hiPSC-CMs

Although there is currently no HCM-specific drug available, β-blockers (class-II antiarrhythmic drug) are commonly prescribed to HCM patients (Spoladore et al., 2012). In this study, bisoprolol was used to investigate whether it would exert antiarrhythmic effects in our hiPSC-CMs. Detailed information about the experimental design for bisoprolol treatment is shown in Fig. S9. In brief, extracellular solution with adrenaline was used prior to the combination of adrenaline and bisoprolol, followed by solution with bisoprolol alone.

#### Role of bisoprolol in HCMT-CMs

**Fig. 7.**
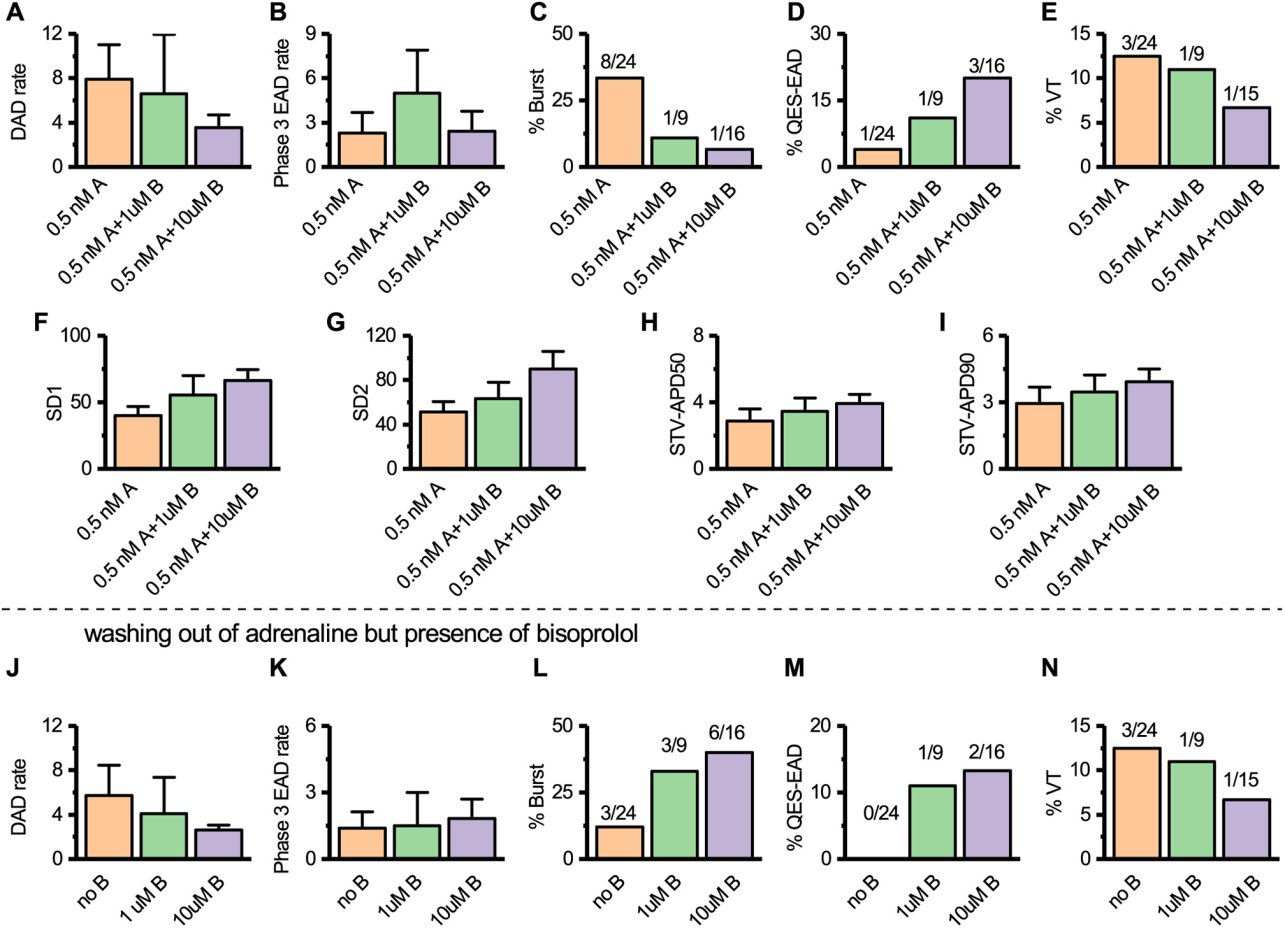
**Treatment of HCMT-CMs with bisoprolol.** (A-E) Quantification of the occurrence of arrhythmia in the presence of 0.5 nM adrenaline (0.5 nM A), with addition of 1 μM (0.5 nM A+1 μM B) and 10 μM (0.5 nM A+10 μM B) bisoprolol. (F-I) Summary of SD1, SD2, STV-APD50 and STV-APD90 in the presence of 0.5 nM adrenaline with addition of 1 μM and 10 μM bisoprolol (*n*=14 for 0.5 nM A, *n*=4 for 0.5 nM A+1 μM B and *n*=7 for 0.5 nM A+10 μM B). (J-N) Quantification of the occurrence of arrhythmia during the wash-out condition of 0.5 nM adrenaline with 0 μM (no B), 1 μM (1 μM B) and 10 μM (10 μM B) bisoprolol (*n*=24 for 0.5 nM A/no B, *n*=9 for 0.5 nM A+1 μM B/1 μM B and *n*=15 for 0.5 nMA+10 μM B/10 μM B).

To examine the efficacy of β-blockers in HCMT-CMs, we chose to administer 0.5 nM adrenaline because all types of arrhythmias were observed at this concentration. At first, we evaluated the potency of 1 µM bisoprolol for 0.5 nM adrenaline in HCMT-CMs. DAD and phase 3 EAD rates were not significantly (*ns*, Kruskal–Wallis, post hoc Dunn test) reduced by bisoprolol in both the presence and wash-out condition of 0.5 nM adrenaline (Fig. 7A,B,J,K). Bursts were reduced by 1 µM bisoprolol in the presence of 0.5 nM adrenaline (Fig. 7C) but not during the wash-out condition of 0.5 nM adrenaline (Fig. 7L). In addition, 1 µM bisoprolol could not reduce the occurrence of QES-EAD in the presence or in the wash-out condition of 0.5 nM adrenaline (Fig. 7D,M). Next, the 10 µM bisoprolol concentration was tested to determine whether a higher concentration of bisoprolol could reduce the arrhythmias. However, 10 µM bisoprolol could not decrease the DAD and phase 3 EAD rates significantly (*ns*, Kruskal–Wallis, post hoc Dunn test), or reduce the occurrence of burst and QES-EAD, in both the presence and wash-out condition of 0.5 nM adrenaline (Fig. 7A-D,J-M). In the presence of either 1 µM or 10 µM bisoprolol, VTs were observed, indicating that bisoprolol failed to prevent the initiation and termination of lethal arrhythmias in HCMT-CMs (Fig. 7E,N). The role of bisoprolol in beat rate variabilities and repolarization variabilities were studied, but no significant changes (*ns*, one-way ANOVA, post hoc Tukey test) were found in the presence of 1 µM or 10 µM bisoprolol (Fig. 7F-I).

#### Role of bisoprolol in HCMM-CMs

**Fig. 8.**
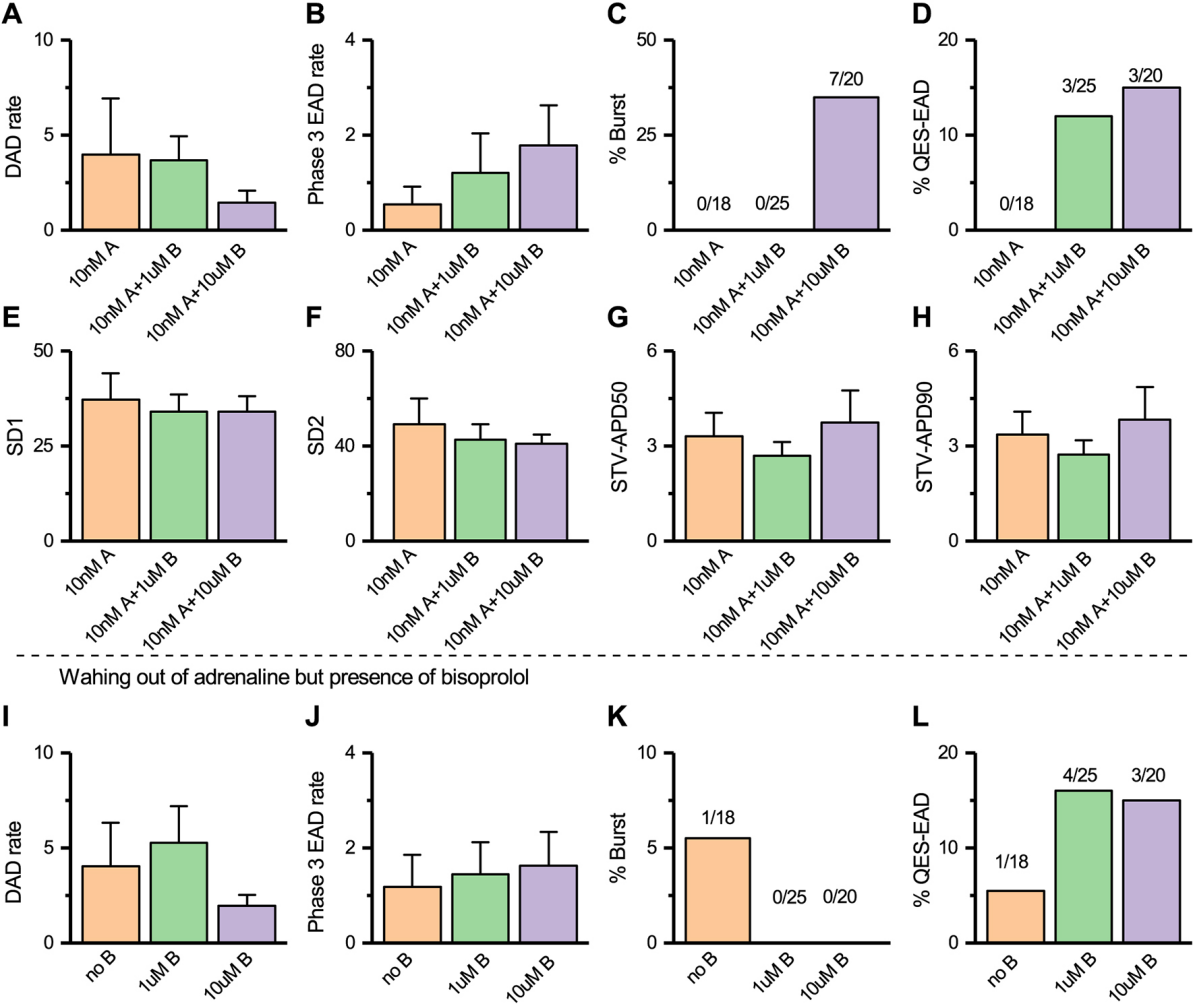
**Treatment of HCMM-CMs with bisoprolol.** (A-D) Quantification of the occurrence of arrhythmia in the presence of 10 nM adrenaline (10 nM A), with addition of 1 μM (10 nM A+1 μM B) and 10 μM (10 nM A+10 μM B) bisoprolol. (E-H) Summary of SD1, SD2, STV-APD50 and STV-APD90 following the administration of 10 nM A, 10 nM A+1 μM B and 10 nM A+10 μM B (*n*=12 for 10 nM A; *n*=17 for 10 nM A+1 μM B; and *n*=12 for 10 μM B). (I-L) Quantification of the occurrence of arrhythmias during wash-out condition of 10 nM A with 0 μM (no B), 1 μM (1 μM B) and 10 μM (10 μM B) bisoprolol (*n*=18 for 10 nM A/no B, *n*=25 for 10 nM A+1 μM B/1 μM B and *n*=15 for 10 nM A+10 μM B/10 μM B).

We also investigated the potency of both 1 µM and 10 µM bisoprolol in the presence and wash-out conditions of 10 nM adrenaline in HCMM-CMs. As shown above, the higher concentrations of adrenaline decreased the occurrence of arrhythmias in HCMM-CMs; thus, a lower concentration of adrenaline was preferred, and the 10 nM adrenaline concentration was chosen. As in HCMT-CMs, neither 1 µM nor 10 µM bisoprolol reduced the DAD rate and phase 3 EAD rate significantly (*ns*, Kruskal–Wallis, post hoc Dunn test) in the presence and wash-out condition of 10 nM adrenaline (Fig. 8A,B,I,J). Burst was not observed in the presence of 1 µM bisoprolol and 10 nM adrenaline and during the wash-out condition of 10 nM adrenaline (Fig. 8C,K). However, bursts were observed in the presence of 10 µM bisoprolol and 10 nM adrenaline, but not during the wash-out condition of 10 nM adrenaline (Fig. 8C,K). Notably, QES-EADs were increased by both 1 µM and 10 µM bisoprolol in the presence and wash-out condition of 10 nM adrenaline (Fig. 8D,L). Furthermore, the beat rate variabilities and repolarization variabilities were not significantly changed (*ns*, one-way ANOVA, post hoc Tukey test) by bisoprolol in HCMM-CMs (Fig. 8E-H).

## DISCUSSION

In our previous study (Ojala et al., 2016), we demonstrated the phenotypic characteristics of HCM in hiPSC-CMs, such as a higher degree of arrhythmias and abnormal calcium handling at baseline conditions. To our knowledge, this is the first study investigating the electrophysiological properties of HCM-specific hiPSC-CMs during the administration and immediately after the wash-out conditions of various concentrations of adrenaline. Furthermore, we investigated the potency of bisoprolol as an antiarrhythmic drug in HCM-specific hiPSC-CMs. Our main findings in the current study are as follows: (1) I_Ca_ and I_to_ are remodeled in HCM-specific hiPSC-CMs; (2) frequencies of ventricular arrhythmias are dependent on concentrations of adrenaline and position of mutation in genes causing HCM; (3) VT types of arrhythmia are observed in HCMT-CMs, but not in HCMM-CMs; and (4) bisoprolol cannot cure arrhythmias in HCM-specific hiPSC-CMs.

### Remodeling of ion channels and arrhythmias

The calcium current densities in both HCM-specific hiPSC-CMs were higher than in WT-CMs, which might explain the prolongation of APD90 in HCM-CMs as shown in our previous study (Ojala et al., 2016). However, in our earlier study, APD90s of HCMT-CMs were longer than those of HCMM-CMs, despite similar I_Ca_ (Ojala et al., 2016). Although I_Ca_ is one of the main components in determining the APD (Han et al., 2014), there are other possible reasons for APD prolongation, such as increased late sodium current (Coppini et al., 2013), decreased rapid-delayed rectifier potassium current (Lahti et al., 2011) and reduced slow-delayed rectifier potassium current (Ma et al., 2015). Interestingly, decreased I_to_ current densities in our HCM-specific hiPSC-CMs was similar to what was observed in isolated cells from HCM patients (Coppini et al., 2013), but this was contradictory to hiPSC-CMs from HCM patients in another study (Han et al., 2014). Furthermore, we found contradictory results in I_K1_ current densities from previous publications, in which isolated cells from HCM patients were used (Coppini et al., 2013). The remodeling of ion channels is considered one of the causes of arrhythmias in HCM (Tomaselli and Marbán, 1999; Knollmann et al., 2003). The characteristics of arrhythmias in HCM have been extensively studied using different models, such as experimental (animal and isolated CMs) (Coppini et al., 2013; Knollmann et al., 2003) and computer models (Passini et al., 2016; Zile and Trayanova, 2017). DAD and EAD could coexist and affect one another because of bidirectional calcium-voltage coupling (Song et al., 2015). The potential mechanisms of DAD (January and Fozzard, 1988), phase 3 EAD (Burashnikov and Antzelevitch, 2003), burst (Chang et al., 2012), long plateau (Song et al., 2015) and VT (Maruyama et al., 2011) types of arrhythmia were described in early studies. Importantly, one distinguished feature observed from the first derivative of all EADs was that the upstroke velocity of EADs was much lower compared to that in APs comprising EAD [Fig. 2, first derivative (Bb,Cb,Db) and phase plots (Bc,Cc,Dc)]. Additionally, phase 3 EAD, burst and QES-EAD arrhythmias recorded during the current clamp might correspond to the double peak, oscillation and plateau types of calcium dynamics, respectively, shown in our earlier study (Ojala et al., 2016). Taken together, these results indicate that these arrhythmias are possibly calcium-driven phenomena.

### Variabilities in hiPSC-CMs

Clinically, the heart rate variabilities in HCM gave mixed results as those parameters depend on age of patients and thickness of left ventricular mass (Counihan et al., 1993; Alter et al., 2006). The variabilities of repolarization indicate a repolarization reserve, suggesting that the higher the variability, the larger the susceptibility for repolarization-dependent ventricular arrhythmias (Varró and Baczkó, 2011). In an early clinical study, the short-term variability (STV) of QT interval was significantly higher in HCM patients than in healthy individuals (Orosz et al., 2015). Another study found that CMs exhibiting ventricular fibrillation (VF) have increased variabilities in APD50 and APD90 than CMs not displaying VF (Sridhar et al., 2008). In our results, VT arrhythmias were only seen in HCMT-CMs, which display significantly higher repolarization variability than WT-CMs. Thus, this indicates the link between repolarization variabilities and VT types of arrhythmias.

### Role of adrenaline in the occurrence of arrhythmias

Most previous studies have focused on isoproterenol for β-adrenergic stimulation and shown an increased amount of arrhythmias in HCM models (Lan et al., 2013; Han et al., 2014; Knollmann et al., 2003). However, we used adrenaline over isoproterenol for β-adrenergic stimulation in this study because adrenaline is an endogenous catecholamine, whereas isoproterenol is an exogenous agent, i.e. not a natural compound. Although isoproterenol only activates the β- (both β1 and β2) adrenergic pathway, adrenaline stimulates both α- and β-adrenergic receptors (Brodde and Michel, 1999). In human, the physiological plasma adrenaline concentration during rest is ∼0.12 nM/l (Omodani et al., 1998; Warren et al., 1984), and adrenaline causes a positive inotropic effect and a decrease in APD90, mainly via the β-adrenergic pathway (Jakob et al., 1988). In contrast to these results, our previous work on catecholaminergic polymorphic ventricular tachycardia (CPVT) using hiPSC-CMs showed that administration of adrenaline (1 µM) increased the arrhythmic episodes in CPVT-specific hiPSC-CMs (Kujala et al., 2012; Penttinen et al., 2015). These conflicting results demonstrate that the different gene mutations inside cardiomyocytes (CMs) give distinct responses, even though the adrenaline levels are the same. Although the *TPM1-Asp175Asn* mutation causing HCM has been considered benign or to represent intermediary risk (Coviello et al., 1997), Hedman and co-workers suggested that HCM patients carrying *TPM1-Asp175Asn* mutation were at increased risk of fatal arrhythmias, and a number of these patients had SCD at a young or middle age, or presented two or three clinical markers for increased risk of SCD [family history of SCD, syncope, NSVT, pathological blood pressure response, marked (>2.5 cm) hypertrophy] (Hedman et al., 2004). On the other hand, the *MYBPC3-Gln1061X* mutation exhibited age-related penetrance with delayed onset of the disease (Jääskeläinen et al., 2002). Moreover, NSVTs were observed in a higher number of HCM patients carrying *MYBPC3-Gln1061X* mutations than HCM patients carrying the *TPM1-Asp175Asn* mutation (23% versus 10%) during 24-h electrocardiogram monitoring (Jääskeläinen et al., 2013). However, in our hiPSC-CM study, we only observed VT types of arrhythmia in HCMT-CMs, thus our finding is in line with earlier clinical studies of patients with the *TPM1-Asp175Asn* mutation being at increased risk of arrhythmia*.* In previous clinical exercise tests, some HCM patients experienced NSVT and VF, and all were asymptomatic (Gimeno et al., 2009). In our study, HCM patients whose hiPSC-CMs showed NSVT are also clinically asymptomatic; however, the NSVT transition to SVT or NRVT occurred only after the administration of adrenaline. Xie et al. (2014) presented a possible mechanism of this transition via β-adrenergic stimulation. Although the occurrence of arrhythmias during exercise is infrequent in HCM, existence of such arrhythmias can be linked to increased risk of SCD (Gimeno et al., 2009). Patel et al. (2014) found that the peak heart rate during exercise testing was lower in HCM patients than in controls. The possible reason for slower heart rate might be reduction in β-adrenergic receptor density (Choudhury et al., 1996) and a desensitized cardiac β-adrenergic system under normal plasma catecholamines in HCM (Schumacher et al., 1995).

### Pharmacology in HCM-specific hiPSC-CMs

Bisoprolol is a selective β1-adrenoceptor blocker (Brixius et al., 2001) and has been used to improve exercise capacity in heart failure (Issa et al., 2007; Dubach et al., 2002). In another clinical study on heart failure, bisoprolol reduced heart rate and increased the heart rate variability parameter (Pousset et al., 1996). Contradictory to our results, earlier studies claimed that β-blockers were able to reduce arrhythmias in HCM-specific hiPSC-CMs (Lan et al., 2013; Han et al., 2014). The possible reasons for these conflicting results might be differences in the study design, including long-term administration versus short-term administration, different concentration and different types of β-adrenergic agonist/antagonist used in our study. Furthermore, we specifically categorized the arrhythmias presented in our hiPSC-CMs, which had not been done in earlier studies (Han et al., 2014; Lan et al., 2013). In addition, an early study comparing antiarrhythmic drug therapy and ICD showed that ICD was superior to antiarrhythmic drugs among patients who experienced VF or sustained VT [Antiarrhythmics versus Implantable Defibrillators (AVID) Investigators, 1997]. The previous study showed that programmed ventricular stimulations (PVS) induced SVT or VF in 33% (7/21) of HCM patients carrying the *TPM1-Asp175Asn* mutation, although 57% (4/7) of those vulnerable HCM patients were under β-blocker therapy (Hedman et al., 2004), indicating that β-blocker treatment is suboptimal in arrhythmia prevention in HCM. Taken together, these results indicate that ICD therapy is the only proven technique for effective termination of VT episode, preventing SCD in high-risk HCM patients (Trivedi and Knight, 2016).

## Study limitations

Although our comprehensive study revealed previously unknown roles of adrenaline and bisoprolol in HCM-specific hiPSC-CMs, it also raises some new questions. Why do hiPSC-CMs with different HCM-causing mutations respond differently to endogenous adrenaline and why are VT types of arrhythmia exclusively presented in hiPSC-CMs with mutation in *TPM1-Asp175Asn*? However, it is beyond the scope of this study to suggest a promising mechanism of the arrhythmias in these hiPSC-CMs. In addition, owing to their developmental immaturity, these hiPSC-CMs exhibit some essential differences from adult CMs, such as lack of t-tubules and low-level expression of IK1 (KCNJ2). Thus, these hiPSC-CMs do not fully resemble adult CMs. Furthermore, hiPSC-CMs do not recapitulate the tissue-level disease phenotype, including interstitial fibrosis and myocardial disarray. Moreover, the human heart develops under the continuous presence of catecholamine in blood and neurointervention, which is certainly not similar in the case of *in vitro* maturation of hiPSC-CMs. Furthermore, the human heart mainly consists of myocytes with other cell types, such as endothelial cells and fibroblasts, and involves crosstalk between the cells. However, hiPSC-CMs lack other cell types and cell-cell communication; thus, microscopic single hiPSC-CMs cannot completely model the whole macroscopic human heart. Moreover, the mutations associated with HCM patients studied herein are the most common mutations in Finland. Therefore, these results might only be specific to these mutations and cannot readily be extrapolated to all HCM patients. Generating the isogenic control cell lines by correcting the HCM-causing mutation with the help of CRISPR/Cas9 to understand the mechanism of HCM diseases in a better way is the aim of future studies.

## Conclusions

This study has clearly shown that while studying β-adrenergic stimulation and blockage in HCM, it is extremely important to address not only during the administration of agonists/antagonist, but also immediately after washing out. Furthermore, the hiPSC-CM model provides a safe and robust platform to study the genetic cardiac diseases. We strongly believe that our results contribute to a better understanding of HCM and create a foundation for future investigation.

## MATERIALS AND METHODS

### Ethical approval

BioMediTech (Heart Group) has received permission from the Ethics Committee of Pirkanmaa Hospital District to conduct research in hiPSCs (R08070). Skin biopsies for hiPSC establishment were received from the Heart Hospital, Tampere University Hospital, Tampere, Finland. The patients donating skin biopsies provided written informed consent.

### Patient-specific hiPSC lines and hiPSC-derived CMs

Seven hiPSC lines were used in this study: three lines with mutation in *TPM1* (*TPM1-Asp175Asn*) (UTA.13602.HCMT, UTA.02912.HCMT and UTA02913.HCMT), two lines with mutation in *MYBPC3* (*MYBPC3-Gln1061X*): (UTA.06108.HCMM and UTA.07801.HCMM), and two lines derived from healthy individuals (UTA.04602.WT and UTA.04511.WT). The characterization of all other hiPSC lines except UTA.02913.HCMT was performed in our previous study (Ojala et al., 2016). Details on hiPSC lines and individuals from whom hiPSC lines were derived are presented in Table S2. The characterization of UTA.02913.HCMT is shown in Fig. S3. hiPSCs were differentiated into CMs (hiPSC-CMs) by co-culturing with mouse visceral endoderm-like cells (END-2) as described in our previous study (Ojala et al., 2016). In this study, 40- to 85-day-old hiPSC-CMs were used for experiments, and control cell lines and cell lines with each mutation were combined in groups. Two hiPSC-CMs derived from two healthy individuals without any known mutations causing HCM were combined to make the WT-CM group. Furthermore, three hiPSC-CMs from two different individuals carrying the same *TPM1* (*TPM1-Asp175Asn*) mutation were combined to make the HCMT-CM group. Similarly, two hiPSC-CMs from two different individuals carrying the same *MYBPC3* (*MYBPC3-Gln1061X*) mutation were combined to make the HCMM-CM group.

### Chemicals and drugs

All drugs and chemicals for electrophysiology experiments were purchased from Sigma-Aldrich (USA) unless otherwise specified. Potassium methanesulfonate (KMeSO_4_) was ordered from MP Biomedicals (USA). (±)-Epinephrine hydrochloride (adrenaline) was dissolved in Milli-Q water, tightly sealed in an Eppendorf tube and stored at −20°C. Cadmium chloride (CdCl_2_) and barium chloride (BaCl_2_) were dissolved in Milli-Q water and stored at 4°C. On the day of experiments, the drugs were diluted to the final concentration in extracellular solution. 4-Aminopyridine (4-AP) was dissolved in the extracellular solution on the day of experiment. Amphotericin-B was first dissolved in dimethyl sulfoxide (DMSO, Sigma-Aldrich) and then added to intracellular solution to a final concentration of 0.24 mg/ml and stored at 4°C during experiments.

### Current clamp

APs were recorded in the gap-free mode from hiPSC-CMs using a perforated patch configuration with amphotericin B as previously described (Ojala et al., 2016). In brief, the hiPSC-CMs were continuously perfused at 36±1°C with an extracellular solution containing 143 mM NaCl, 4.8 mM KCl, 1.8 mM CaCl_2_, 1.2 mM MgCl_2_, 5 mM glucose and 10 mM HEPES (pH was adjusted to 7.4 with NaOH). The intracellular solution contained 132 mM KMeSO_4_, 20 mM KCl, 1 mM MgCl_2_, 4 mM EGTA and 1 mM CaCl_2_ (pH was adjusted to 7.2 with KOH).

### Voltage clamp

Once the stable baseline APs were obtained, the amplifier was switched into voltage clamp mode to record ionic currents maintaining the same extracellular solution. Ionic currents were divided by cell capacitances and presented as pA/pF. All measurements were performed at 36±1°C. The calcium current (I_Ca_) was measured with a depolarizing potential of −50 mV to 80 mV with 10 mV increments. The holding potential (HP) was −40 mV to inactivate the sodium channels, and 3 mM 4-AP was used in the extracellular solution to block the transient outward potassium current (I_to_). Early study showed that there was less contamination from sodium ions in the calcium current measurement (Fatima et al., 2013), I_to_ was sodium dependent, and either removing or blocking sodium ions reduced the peak I_to_ current (Dukes and Morad, 1991). Thus, sodium ions were not removed and sodium channels were not blocked in this study. Next, I_to_ was measured with the two-step protocol from a HP of −80 mV: a first step of −50 mV was used to inactivate the sodium channels and then to test potentials from −50 mV to 70 mV with a step size of 10 mV. We added 300 µM CdCl_2_ to the extracellular solution to block the calcium currents. The inward rectifier potassium current (I_K1_) was measured with 2 mM BaCl_2_-sensitive current in the presence of 300 µM CdCl_2_ and 3 mM 4-AP. The currents were elicited by a depolarizing potential of −140 mV to 0 mV with 10 mV increments, and HP was −40 mV. In addition, the I_K1_ current was extracted by the subtraction of currents recorded with and without the presence of BaCl_2_ in the extracellular solution.

### Variability analysis

Beat rate variability (BRV) was assessed from ≥30 consecutive APs without the presence of arrhythmias. SD1 is the standard deviation (SD) of the instantaneous (STV) beat-to-beat interval variability. SD2 is the SD of the long-term interval variability (LTV). SD1 and SD2 were calculated using the formulae SD1^2^=1/2×SDSD^2^ and SD2^2^=2SDRR^2^−1/2×SDSD^2^, respectively, in which SDRR represents the SD of all RR intervals and SDSD represents the SD of differences between adjacent RR intervals. Similarly, the STV-APD50 and STV-APD90 were assessed in the same APs selected for BRV using the formulae 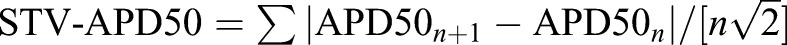 and 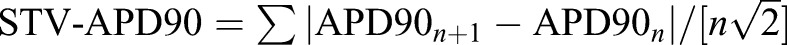, respectively. *n* represents the number of APs used to calculate variabilities.

### Data analysis

Recorded APs were analyzed using custom-made software in Origin9.1 (OriginLab Corp., USA) to extract BPM, APD50 and APD90, APA, dV/dt and MDP. The recorded ionic currents were analyzed using Clampfit 10.7 (Molecular Devices, USA). The SD1, SD2, SDRR, SDSD, STV-APD50 and STV-APD90 were calculated using custom-made software in Matlab R2015a (MathWorks, Inc., USA).

### Statistical analysis

For independent group comparisons, a one-way ANOVA followed by Tukey's post hoc test was used for normally distributed data. In addition, a paired *t*-test was used for the dependent normally distributed data. For the three independent variables, the Kruskal–Wallis test followed by the Dunn test was used for non-normally distributed data. For the three dependent variables, the Friedman test followed by the Dunn test was used for non-normally distributed data. A two-tailed *P*<0.05 was considered statistically significant. Data are presented as mean±standard error of mean (s.e.m.).

## Supplementary Material

Supplementary information
