# Supplementary material for: Divergent effects of adrenaline in human induced pluripotent stem cell-derived cardiomyocytes obtained from hypertrophic cardiomyopathy
Source: Dis Model Mech. 2018 Feb 1;11(2):dmm032896. doi: 10.1242/dmm.032896 (PMC5894949; doi:10.1242/dmm.032896)
Supplement: Supplementary information [file dmm-11-032896-s1.pdf]

Supplementary Information

Table S1. Summary of action potential properties of atrial-like hiPSC-CMs.

	Beats rate (BPM)	APD_{50} (ms)	APD_{90} (ms)	APA_{Max} (mV)	MDP (mV)	V_{max} (V/S)
WT-CMs (21)	87.2 ± 6.9	110.2± 13.9	166.0±17.6	110.4 ± 2.5	-74.6 ±1.3	73.9 ± 10.8
HCMT-CMs (30)	75.2 ± 4.9	155.6±13.0	225.1±18.3	108.2±2.1	-71.9±1.0	63.3 ±9.1
HCMM-CMs (34)	76.6±3.0	141.8±9.6	210.4±14.4	112.1±2.0	-74.0±0.9	65.9±6.9

Data are presented as mean±SEM. No significant differences were found in any parameters among groups (*ns*, one-way ANOVA, post hoc Tukey test).

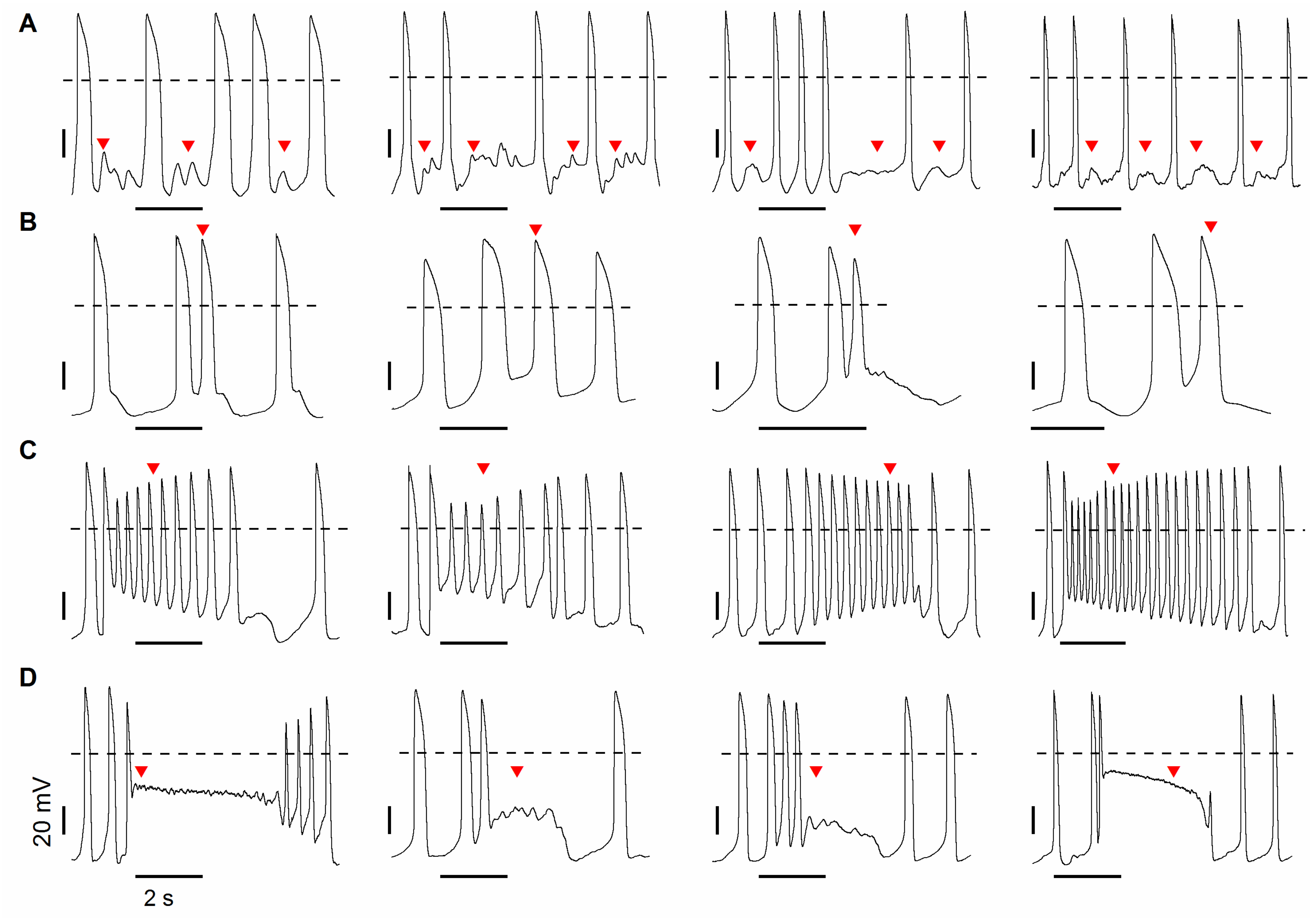

Disease Models & Mechanisms • Supplementary information

Figure S1. Various types of arrhythmias recorded in ventricular-like hiPSC-CMs**.** (A) Representative APs with single and multiple DADs (B) Representative APs with different amplitude and APDs of Phase 3 EAD (C) Representative APs with increasing and decreasing APA in burst EAD (D) Representative APs with varying plateau potential in QES-EAD. Dashed line represent 0 mV.

Disease Models & Mechanisms • Supplementary information

Table S2: Detailed information of hiPSC lines, mutations and individuals from which hiPSC lines were derived.

Cell line	Reprogramming method	Mutation	Sex	Age	IVS (mm)	Other symptoms	SCD in family	Treatment
**UTA.04602.WT**	Retrovirus		F	56	--			
**UTA.04511.WT**	Sendai virus		M	34	--			
**UTA.13602.HCMT**	Sendai virus	*TPM1-Asp175Asn*	F	48	16	Collapsed when 20 years old (normal heart structure)	mother at age of 51	none
**UTA.02912.HCMT**<br>**UTA.02913.HCMT**	Sendai virus	*TPM1-Asp175Asn*	M	33	26	Asymptomatic	One family member at age of 21	β-blocker
**UTA.06108.HCMM**	Retrovirus	*MYBPC3-Gln1061X*	M	55	22	Asymptomatic	father at age of 36<br>uncle at age of 38	none
**UTA.07801.HCMM**	Retrovirus (Cre-LoxP	*MYBPC3-Gln1061X*	M	61	25	Atrial fibrillation	none	β-blocker<br>ICD

IVS: Intraventricular septum

SCD: Sudden cardiac death

ICD: Implantable cardioverter defibrillator

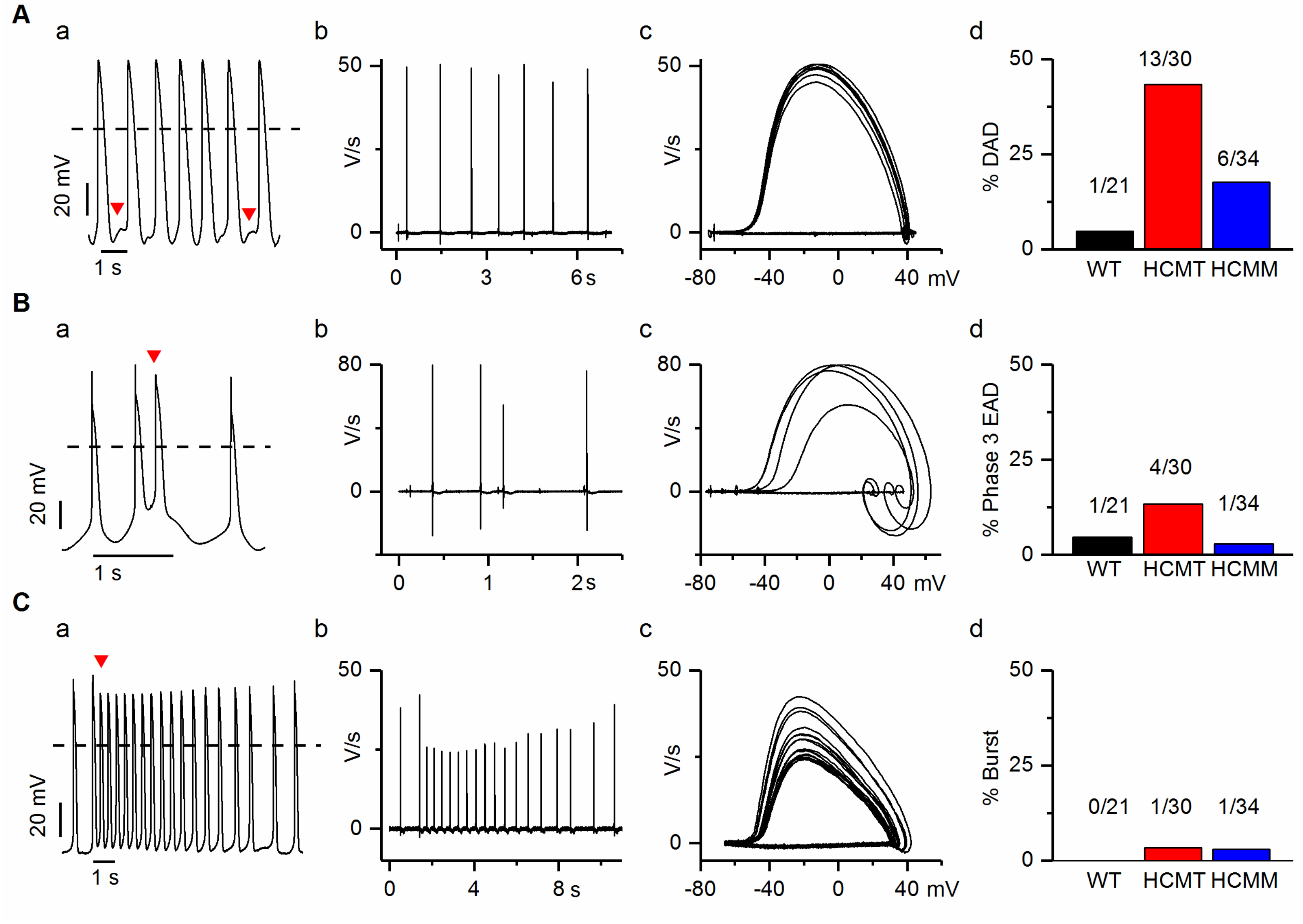
A
a
20 mV
1 s
b
50
V/s
0
0
3
6 s
c
50
V/s
0
-80
-40
0
40 mV
d
50
25
0
% DAD
13/30
1/21
6/34
WT
HCMT
HCMM
B
a
20 mV
1 s
b
80
V/s
0
0
1
2 s
c
80
V/s
0
-80
-40
0
40 mV
d
50
25
0
% Phase 3 EAD
4/30
1/21
1/34
WT
HCMT
HCMM
C
a
20 mV
1 s
b
50
V/s
0
0
4
8 s
c
50
V/s
0
-80
-40
0
40 mV
d
50
25
0
% Burst
0/21
1/30
1/34
WT
HCMT
HCMM

Figure S2. Summary of baseline characteristics of atrial-like hiPSC-CMs. Representative APs with presence of DADs (Aa) its first derivative (Ab) its phase plot (Ac) and percentage of cells exhibiting DADs (Ad) Representative APs with phase 3 EAD (Ba) its first derivative (Bb) its phase plot (Bc) and percentage of cells exhibiting Phase 3 EAD (Bd), Representative APs with burst (Ca) its first derivative (Cb) its phase plot (Cc) and percentage of cells exhibiting Burst (Cd). Respective arrhythmias are indicated by an arrow (▼).

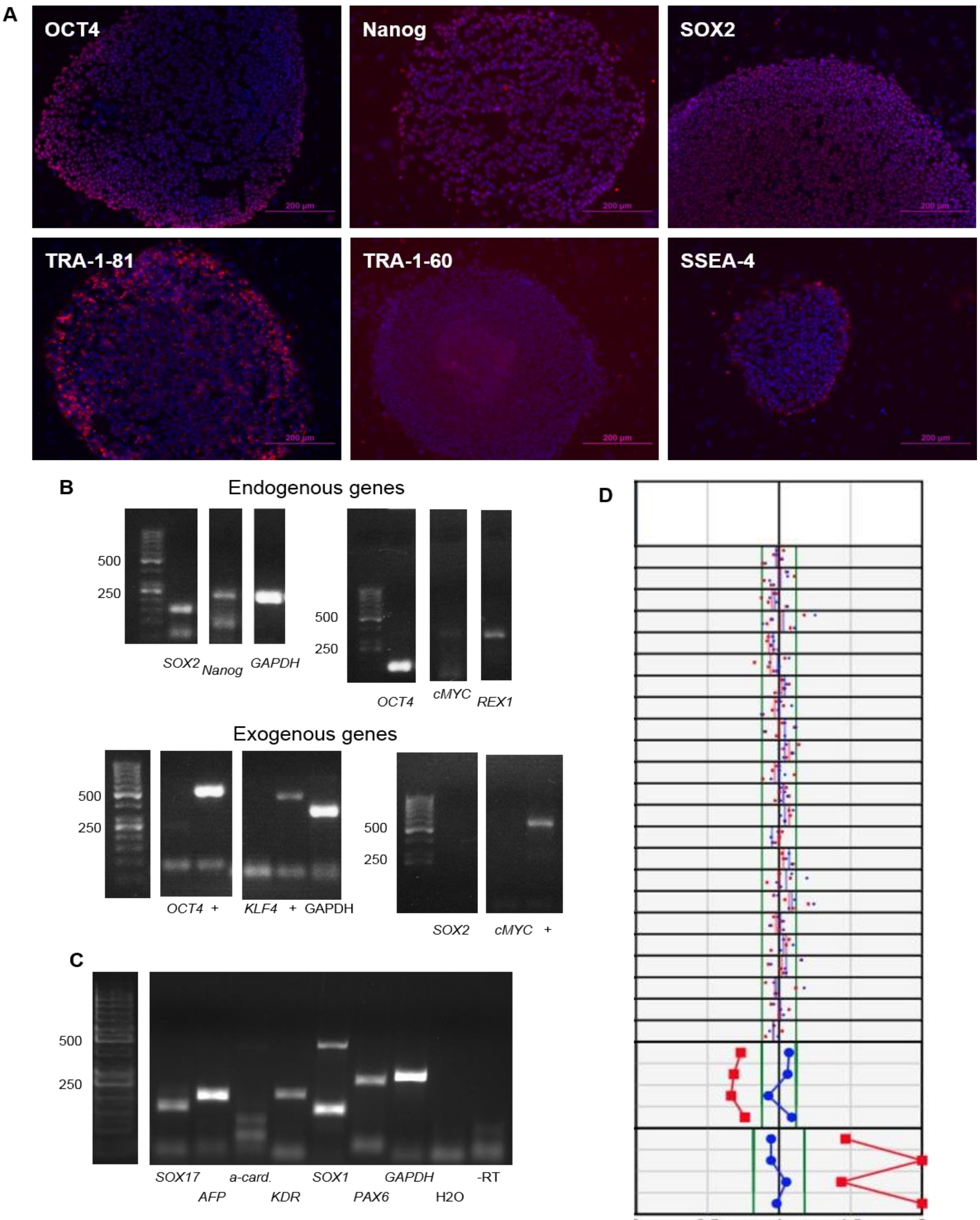

Figure S3. Characterization of UTA.02913.HCMT hiPSC line. (A) The hiPSCs formed colonies expressing Oct4, Nanog, Sox2, TRA-1-81, TRA-1-60 and SSEA-4. (B) hiPSCs expressed endogenous *SOX2* (151bp), *Nanog* (287bp), *OCT4* (144bp), *cMYC* (328bp) and *REX1* (306bp). Virally transferred Sendai exogenes *exo-OCT4* (483bp), *exo-KLF4* (410bp), *exo-SOX2* (451bp) and *exo-cMYC* (532bp) were absent in hiPSCs. + indicates positive controls. (C) As a proof of pluripotency, hiPSCs were differentiated into EBs, which expressed markers from all germ layers: endoderm (*SOX17* (120bp), *AFP* (209bp)), mesoderm (*α-cardiac actin* (486bp), *KDR* (218bp)) and ectoderm (*SOX1* (166bp), *PAX6* (274bp)). *GAPDH* (302bp) was used as a housekeeping control in each PCR experiment. (D) The hiPSC line was karyotypically normal (46, XY) in KaryoLite BoBs Assay (PerkinElmer).

Disease Models & Mechanisms • Supplementary information

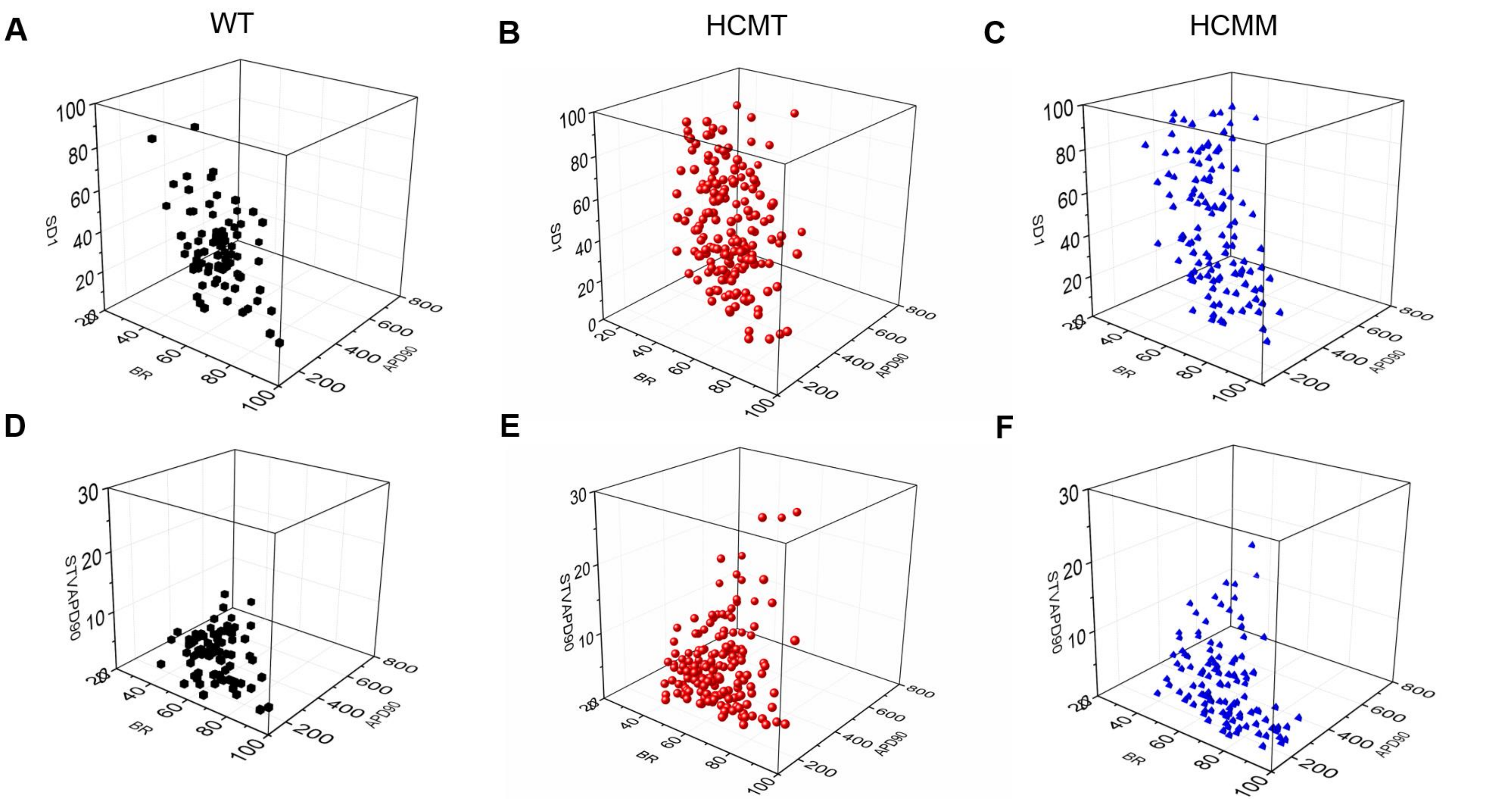

Figure S4: Three-dimensional representation of beat rate and repolarization variabiltites and AP parameters. (A-C) 3-D plot of beat rate, APD90 and SD1 and (D-F) beat rate, APD90 and STV-APD90 in WT-CMs (n=79, ■), HCMT-CMs (n=174, ●) and HCMM-CMs (n=118, ▲).

Disease Models & Mechanisms • Supplementary information

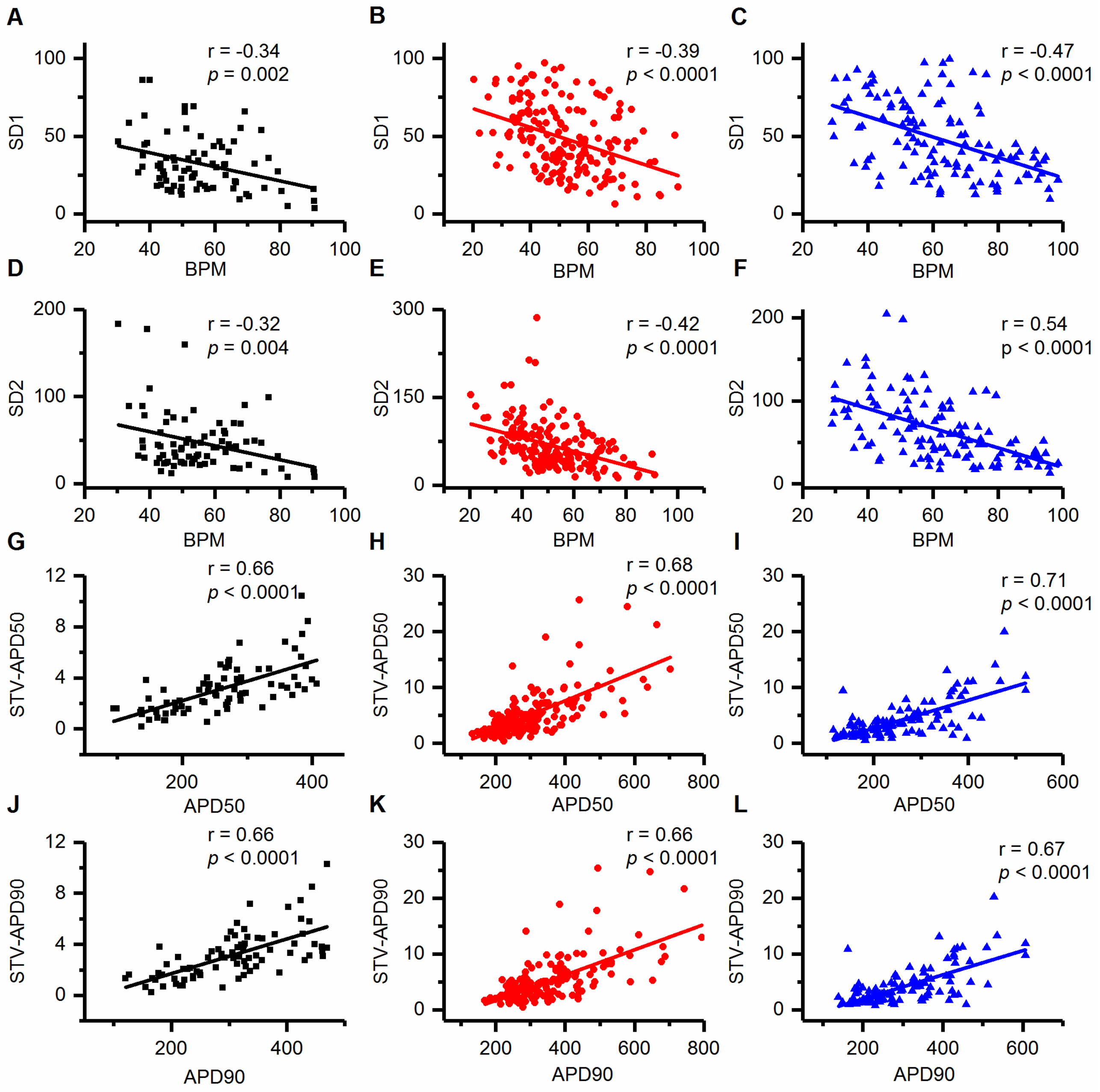

Figure S5. Beat rate and repolarization variabilities parameters as a function of BPM, APD50 and APD90. Correlation between beat rate variabilities with their beat rates and APD variabilities with their APDs in WT-CMs (n=79, ■), HCMT-CMs (n=174, ●) and HCMM-CMs (n=118, ▲). 'r' represents the correlation coefficient where '–' sign means inverse relationship and vice versa

Disease Models & Mechanisms • Supplementary information

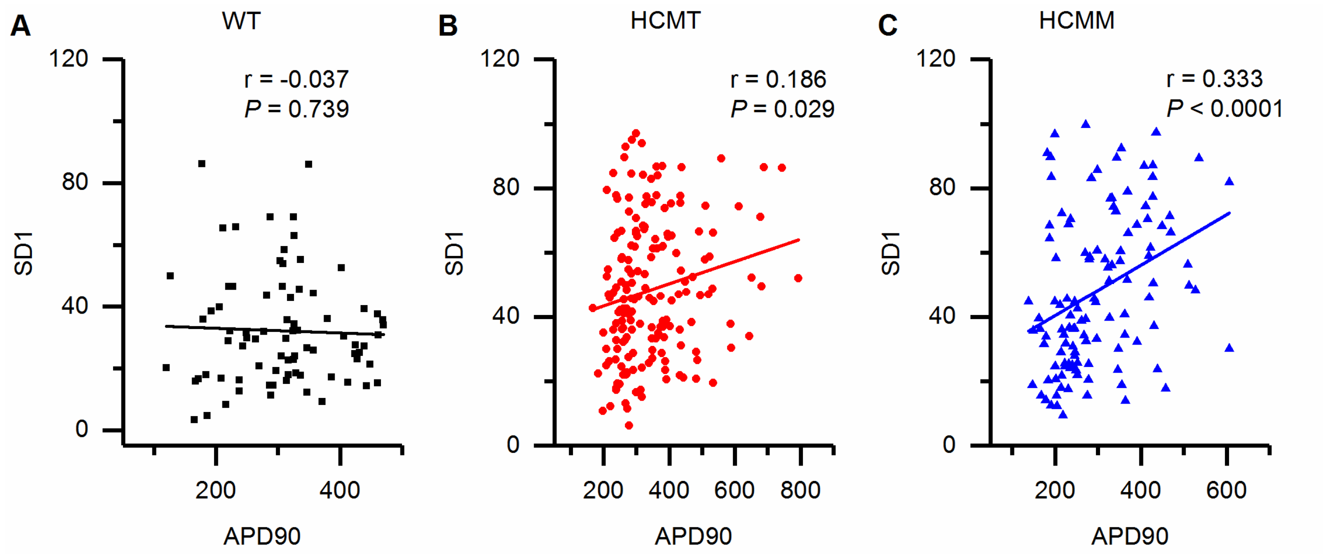

Figure S6. Graph of SD1 vs. APD90. Correlation between SD1 and APD90 in WT-CMs (n=79, ■), HCMT-CMs (n=174, ●) and HCMM-CMs (n=118, ▲). No correlation between SD1 and APD90 was found in WT-CMs.

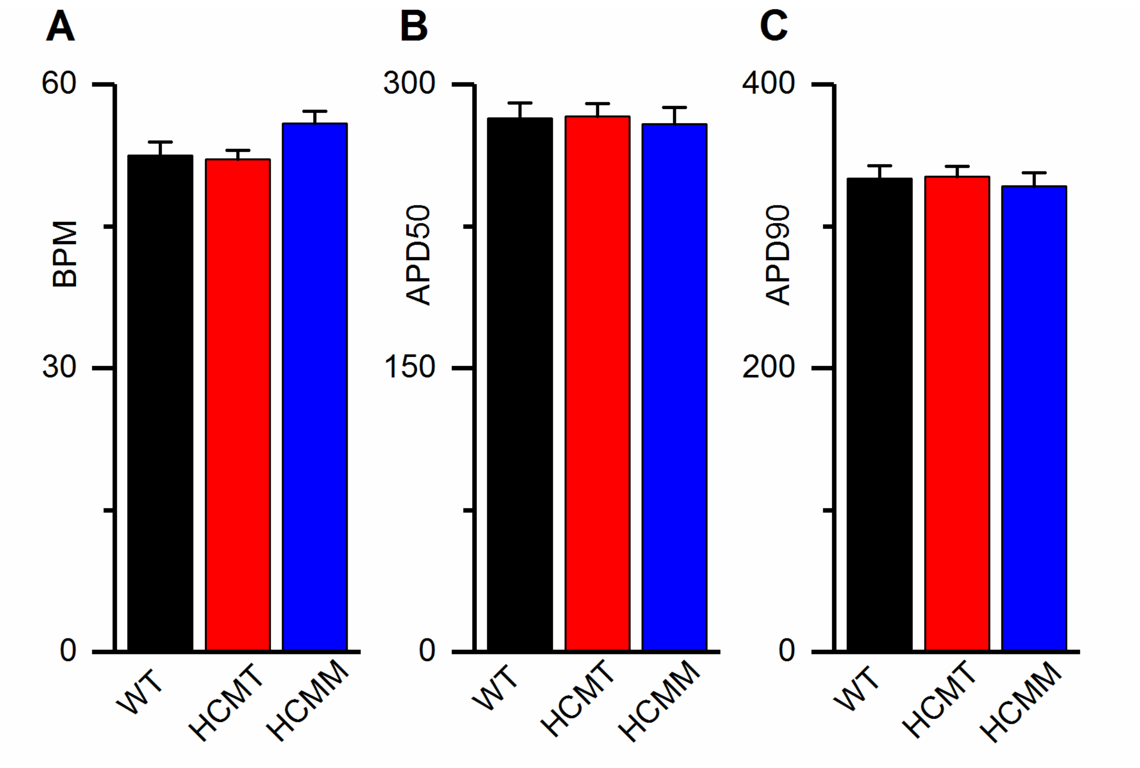

Figure S7. Comparison of BPM, APD50 and APD90. Summary of hiPSC-CMs fulfilling the conditions of 30 to 60 bpm, and APD of 200 to 600 ms in WT-CMs (n=63), HCMT-CMs (n=154) and HCMM-CMs (n=119). No significant differences were found among the groups. (*ns*, one-way ANOVA, post hoc Tukey test)

Disease Models & Mechanisms • Supplementary information

Table S3. Summary of variabilities in atrial-like hiPSC-CMs.

	SD1	SD2	SDRR	SDSD	SV_ADP90	SV_APD50
WT-CMs (21)	29.6±3.9	36.9±6.3	33.7±5.1	41.9±5.5	1.6±0.2	1.2±0.1
HCMT-CMs (27)	29.1±2.9	38.0±3.6	34.1±3.2	41.2±4.2	1.9±0.2	1.7±0.2
HCMM-CMs (33)	27.1±5.5	33.0±4.0	30.4±3.7	38.3±5.0	2.0±0.3	1.7±0.2

Data are presented as mean±SEM. No significant differences were found in any parameters among groups (*ns*, one-way ANOVA, post hoc Tukey test)

Disease Models & Mechanisms • Supplementary information

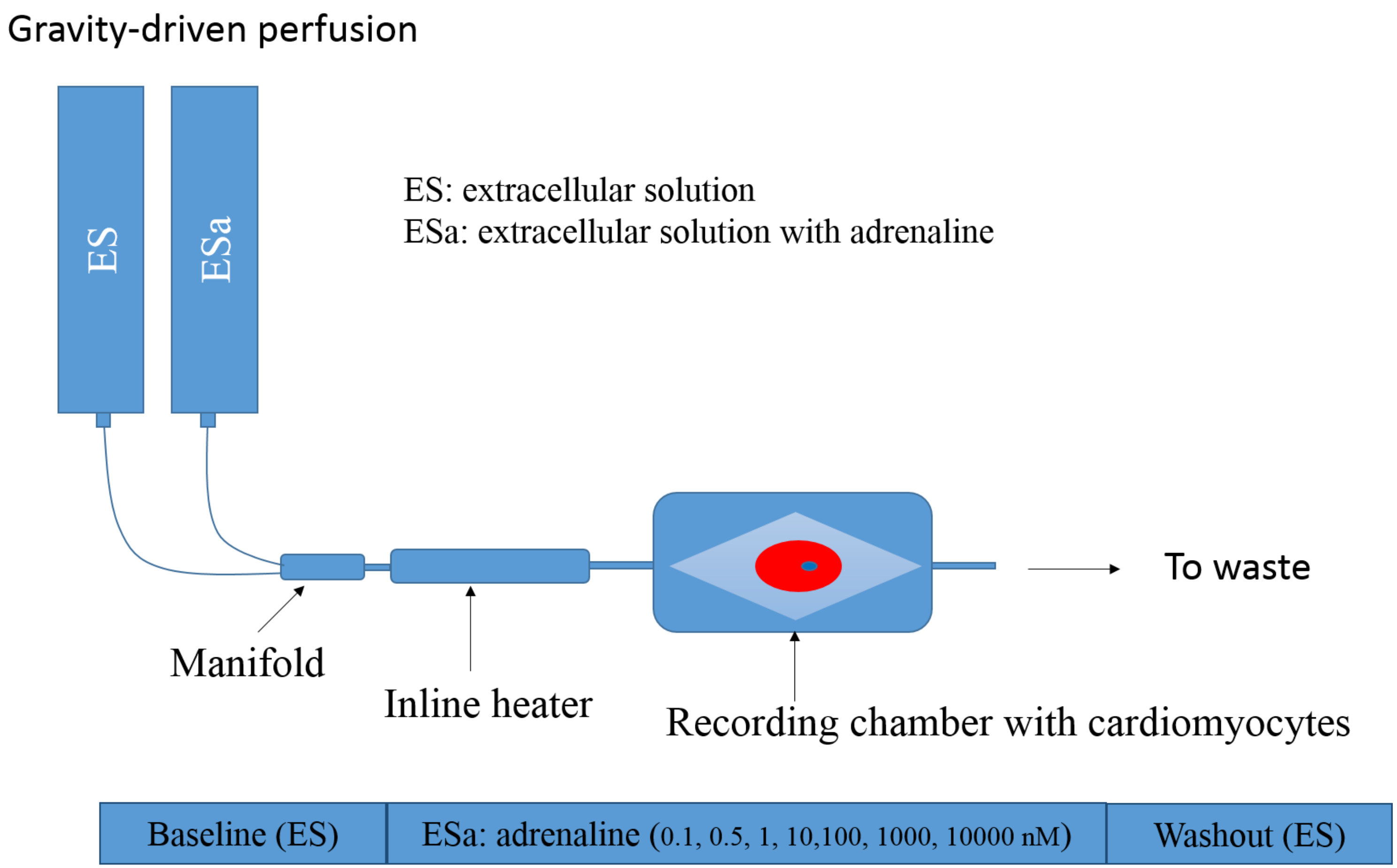

Figure S8. Schematic diagram of experimental setup and protocol used for adrenaline testing**.** Once the stable baseline was recorded with ES, adrenaline with one concentration (ESa) at a time was infused. Adrenaline was passed though the cells for minimum of 4 min, after that extracellular without adrenaline (ES) was passed through cells to wash out adrenaline from the recording chamber. Stable APs were selected for baseline characteristics. APs after minimum of 2 min of adrenaline infusion were selected to study the effect of adrenaline. Furthermore, to study the effect of adrenaline in washing out condition, maximum up to 300ms immediately after the adrenaline being washout were selected for analysis.

Disease Models & Mechanisms • Supplementary information

Table S4. Percentage change in AP characteristic in WT-CMs at different concentrations of adrenaline.

**Adrenaline (nM)**	**Δ%BPM**	**Δ%APD90**	**Δ%APD50**	**Δ%APA**	**Δ%MDP**	**Δ%dVdT**
**0.1** (n=13)	4.99±1.38**	-10.0±1.37***	-7.78±1.09***	-0.41±0.71	-0.61±0.75	1.04±4.36
**0.5** (n=10)	10.09±1.89***	-7.97±0.78***	-5.99±0.84***	0.15±0.36	-0.43±0.57	11.36±5.06
**1** (n=17)	11.71±1.38***	-11.27±1.32***	-9.57±1.27***	-1.02±0.47*	-1.09±0.62	3.67±2.91
**10** (n=8)	40.66±4.2***	-17.57±2.53**	-17.94±3.08**	-1.32±0.69	-1.52±1.25	4.35±6.35
**100** (n=12)	47.67±4.94***	-14.53±1.99**	-15.30±2.11**	-3.29±0.89**	-2.91±0.77**	-14.72±10.98
**1000** (n=11)	59.60±9.42***	-17.38±1.94***	-17.46±1.96***	-1.61±0.64*	-0.54±0.60	-4.42±9.05
**10000** (n=14)	68.67±8.29***	-22.44±2.64***	-24.47±2.88***	-5.51±0.69***	-4.31±0.63***	0.02±7.28

Data presented as mean±SEM. * $P < 0.05$, ** $P < 0.005$ and *** $P < 0.0001$ (paired *t*-test, pre adrenaline vs. post adrenaline). The '–' sign means that the value had decreased from its corresponding pre-drug administration. n represents the number of cells used in each experiment

Disease Models & Mechanisms • Supplementary information

Table S5. Percentage change in AP characteristic in HCMT-CMs at different concentrations of adrenaline.

Adrenaline (nM)	Δ%BPM	Δ%APD90	Δ%APD50	Δ%APA	Δ%MDP	Δ%dVdT
**0.1** (n=29)	7.73±1.10***	-6.28±1.11***	-5.16±0.98***	-0.93±0.41*	-0.78±0.52	-3.73±2.74
**0.5** (n=22)	14.53±1.61***	-6.29±1.39***	-5.80±1.30***	-1.26±0.52*	-1.14±0.60***	-2.77±4.33
**1** (n=32)	17.28±2.31***	-7.32±1.04***	-6.59±1.05***	-1.63±0.50**	-2.15±0.55***	-1.64±3.43
**10** (n=30)	33.01±3.63***	-9.15±1.28***	-8.73±1.20***	-2.68±0.67***	-3.86±0.89***	-5.86±5.25
**100** (n=19)	59.51±5.23***	-18.37±1.80***	-19.01±1.93***	-5.50±0.83***	-1.44±0.65*	-6.36±6.93
**1000** (n=24)	40.29±3.96***	-14.50±1.96***	-14.84±2.02***	-4.29±0.57***	-2.95±0.44***	-2.99±4.21
**10000** (n=29)	49.74±3.67***	-15.14±1.74***	-15.44±2.03***	-4.20±0.66***	-4.56±0.82***	2.42±4.48

Data presented as mean±SEM. * $P < 0.05$, ** $P < 0.005$ and *** $P < 0.0001$ (paired *t*-test, pre adrenaline vs. post adrenaline). The '–' sign means that the value had decreased from its corresponding pre-drug administration. n represents the number of cells used in each experiment

Disease Models & Mechanisms • Supplementary information

Table S6. Percentage change in AP characteristic in HCMM-CMs at different concentration of adrenaline.

Adrenaline (nM)	BPM	APD90	APD50	APA	MDP	dVdT
**0.1** (n=14)	8.09±2.16**	-7.48±1.68**	-6.48±1.24***	-0.27±0.35	0.10±0.46	3.68±3.96
**0.5** (n=15)	15.46±3.48**	-7.87±1.57***	-7.62±1.46***	-1.08±0.52*	-0.70±0.63	-3.75±3.60
**1** (n=15)	24.85±3.78***	-11.43±1.65***	-11.52±1.6 ***	-0.15±0.43	-0.88±0.78	-2.27±6.24
**10** (n=18)	34.37±4.81***	-21.04±1.80***	-21.72±2.06***	-4.45±0.92***	-2.42±1.07*	-5.26±4.80
**100** (n=10)	34.45±5.67***	-11.30±3.21*	-11.98±3.62*	-4.34±0.91***	-1.73±0.46**	3.60±3.92
**1000** (n=10)	37.44±9.95**	-11.96±3.67*	-12.53±3.90*	-3.45±0.91**	-1.22±1.08	-1.08±9.33
**10000** (n=14)	46.18±8.8***	-17.28±2.34***	-17.94±2.49***	-4.17±0.77***	-2.07±0.79*	-13.58±5.60

Data presented as mean±SEM. * $P < 0.05$, ** $P < 0.005$ and *** $P < 0.0001$ (paired *t*-test, pre adrenaline vs. post adrenaline). The '–' sign means that the value had decreased from its corresponding pre drug administration. n represents the number of cells used in each experiment.

Disease Models & Mechanisms • Supplementary information

Table S7. Correlation between BPM and APD90 and their corresponding percentage changes at different concentrations of adrenaline.

	WT-CMs			HCMT-CMs			HCMM-CMs		
**nM**	**n**	**Δ%BPM**	**Δ%APD90**	**n**	**Δ%BPM**	**Δ%APD90**	**n**	**Δ%BPM**	**Δ%APD90**
**0.1**	13	4.99±1.38	10.0±1.37	29	7.73±1.10	6.28±1.11<br># r = -0.3807	14	8.09±2.16	7.48±1.68
**0.5**	10	10.09±1.89	7.97±0.78	22	14.53±1.61	6.29±1.39	15	15.46±3.48	7.87±1.57
**1**	17	11.71±1.38	11.27±1.32	32	17.28±2.31	7.32±1.04<br>** r = 0.5184	15	24.85±3.78<br>## r = -0.6938	11.43±1.65<br>* r = 0.5961
**10**	8	40.66±4.2	17.57±2.53	30	33.01±3.63<br>## r = -0.5037	9.15±1.28<br># r = -0.4371	18	34.37±4.81	21.04±1.80
**100**	12	47.67±4.94<br># r = -0.7259	14.53±1.99	19	59.51±5.23	18.37±1.80	10	34.45±5.67<br># r = -0.6630	11.30±3.21
**1000**	11	59.60±9.42<br># r = -0.6157	17.38±1.94	24	40.29±3.96	14.50±1.96<br>## r = -0.6084	10	37.44±9.95	11.96±3.67
**10000**	14	68.67±8.29<br># r = -0.6304	22.44±2.64	29	49.74±3.67<br>## r = -0.4942	15.14±1.74<br>** r = 0.5309	14	46.18±8.8	17.28±2.34<br>** r = 0.6220

Data are presented as mean±SEM. * $P < 0.05$, ** $P < 0.005$ (Pearson's correlation test between Δ% BPM vs. Δ% APD90) and # $P < 0.05$, ## $P < 0.005$ (Pearson's correlation test between BPM vs. Δ% BPM or APD90 vs. Δ% APD90). 'r' represents the correlation coefficient where '–' sign means inverse relationship and vice versa. n represent the number of cells used in each experiment.

Table S8. DAD rate (1/min) in hiPSC-CMs. Frequency of DADs was calculated as total number of DADs/total number of APs.

	WT-CMs				HCMT-CMs				HCMM-CMs			
**nM**	**n**	**Baseline**	**Adrenaline**	**Washout**	**n**	**Baseline**	**Adrenaline**	**Washout**	**n**	**Baseline**	**Adrenaline**	**Washout**
**0.1**	13	0.29±0.20	0.89±0.34	1.0±0.74	29	7.16±2.26**	6.85±2.18	6.24±2.27	14	4.59±1.65	3.72±1.38	2.08±1.08
**0.5**	10	0.5±0.15	0.73±0.66	1.26±0.73	24	6.41±2.78	7.93±3.1	5.74±2.72	15	3.9±1.98	4.69±2.18	3.09±1.93
**1**	17	1.5±0.8	2.0±1.13	2.4±1.0	32	6.19±2.49	5.3±1.81	5.09±1.73	15	6.6±2.10	4.35±1.61	4.05±2.05
**10**	8	0.0±0.0	0.0±0.0	0.0±0.0	31	6.36±2.16*	5.72±1.86*	4.0±1.72	18	1.78±0.53#	3.97±2.8	4.03±2.18
**100**	12	1.43±1.3	0.60±0.42	0.24±0.17	19	2.26±0.98	0.97±0.9	1.27±0.7	11	2.98±1.17	1.23±0.72	2.08±0.96
**1000**	11	0.98±0.7	0.37±0.35	0.55±0.48	24	2.75±1.05	1.65±0.7	2.48±1.21	10	5.65±2.77	1.18±0.60	0.94±0.52
**10000**	14	1.09±0.6	0.0±0.0	0.0±0.0	29	3.53±1.19	1.56±0.57**	2.07±0.63**	14	3.33±1.19	1.43±0.65	1.34±0.75

DAD rates did not change significantly during and immediately after administration of different concentrations of adrenaline from baseline in WT-CMs, HCMT-CMs and HCMM-CMs. (*ns*: Friedman, posthoc Dunn test). * or # P<0.05, **P<0.005 (WT-CMs Vs HCMT-CMs or HCMM-CMs; Kruskal Wallis, post hoc Dunn test). Data are presented as mean±SEM. n represents the number of cells used in each experiment.

Disease Models & Mechanisms • Supplementary information

Table S9. Phase 3 EAD rate (1/10min) in hiPSC-CMs. Frequency of phase 3 EAD rate was calculated as total number of Phase 3 EAD/total time.

	WT-CMs				HCMT-CMs				HCMM-CMs			
**nM**	**n**	**Baseline**	**Adrenaline**	**Washout**	**n**	**Baseline**	**Adrenaline**	**Washout**	**n**	**Baseline**	**Adrenaline**	**Washout**
**0.1**	13	0.0±0.0	0.7±0.4	0.8±0.8	29	0.1±0.1	3.8±2.5	2.1±1.2	14	0.3±0.3	0.5±0.3	0.2±0.2
**0.5**	10	0.0±0.0	0.3±0.3	0.0±0.0	24	3.8±1.7	2.3±1.4	1.4±0.7	15	0.6±0.6	1.5±0.9	0.0±0.0
**1**	17	0.2±0.2	1.0±0.6	0.0±0.0	32	1.6±0.5	10.0±4.8	7.7±3.6*	15	1.0±1.0	0.7±0.4	0.9±0.5
**10**	8	0.0±0.0	0.0±0.0	0.5±0.5	31	12.9±8.7	14.6±11.7	7.2±6.5	18	0.3±0.3	0.5±0.4	1.2±0.6
**100**	12	0.3±0.3	0.0±0.0	0.0±0.0	19	2.0±1.2	0.7±0.5	1.0±0.6	11	0.9±0.9	0.7±0.5	0.2±0.2
**1000**	11	0.4±0.4	0.0±0.0	0.0±0.0	24	0.8±0.6	0.9±0.5	1.0±0.5	10	2.6±1.1	1.2±0.8	1.8±0.7$^{\#}$
**10000**	14	0.0±0.0	0.0±0.0	0.0±0.0	29	0.5±0.3*	1.1±0.5	1.1±0.7	14	3.9±1.5$^{\#}$	0.5±0.3	1.1±0.5

Phase 3 EAD rate did not change significantly during and immediately after administration of different concentrations of adrenaline from baseline in WT-CMs, HCMT-CMs and HCMM-CMs. (*ns*: Friedman, post hoc Dunn test). * or # P<0.05 (WT-CMs Vs HCMT-CMs or HCMM-CMs; Kruskal Wallis, post hoc Dunn test). Data are presented as mean±SEM. n represents the number of cells used in each experiment.

Table S10. Categorical analysis of occurrence of burst in hiPSC-CMs. Frequency of burst was calculated as total number of burst/total number of cells.

	**WT-CMs**				**HCMT-CMs**				**HCMM-CMs**			
**nM**	**n**	**Baseline**	**Adrenaline**	**Washout**	**n**	**Baseline**	**Adrenaline**	**Washout**	**n**	**Baseline**	**Adrenaline**	**Washout**
**0.1**	13	23% (3)	8% (1)	0% (0)	29	0% (0)	7% (2)	3% (1)	14	7% (1)	14% (2)	7% (1)
**0.5**	10	0% (0)	10% (1)	0% (0)	24	13% (3)	38% (9)	13% (3)	15	7% (1)	13% (2)	7% (1)
**1**	17	12% (2)	18% (3)	12% (2)	32	0% (0)	28% (9)	44% (14)	15	0% (0)	13% (2)	20% (3)
**10**	8	0% (0)	0% (0)	13% (1)	31	26% (8)	13% (4)	6% (2)	18	17% (3)	0% (0)	6% (1)
**100**	12	0% (0)	0% (0)	0% (0)	19	5% (1)	5% (1)	21% (4)	11	0% (0)	0% (0)	0% (0)
**1000**	11	0% (0)	0% (0)	0% (0)	24	0% (0)	4% (1)	13% (3)	10	0% (0)	0% (0)	0% (0)
**10000**	14	7% (1)	0% (0)	0% (0)	29	7% (2)	7% (2)	3% (1)	14	14% (2)	0% (0)	7% (1)

Data are presented as percentages. The percentages were rounded off to the whole number. n represents the number of cells used in each experiment. The value in parenthesis represents the number of burst occurred in each experiment.

Disease Models & Mechanisms • Supplementary information

Table S11. Occurrence of QES-EAD in hiPSC-CMs. Frequency of QES-EAD was calculated as total number of QES-EAD/total number of cells.

	WT-CMs				HCMT-CMs				HCMM-CMs			
**nM**	**n**	**Baseline**	**Adrenaline**	**Washout**	**n**	**Baseline**	**Adrenaline**	**Washout**	**n**	**Baseline**	**Adrenaline**	**Washout**
**0.1**	13	0% (0)	8% (1)	8% (1)	29	0% (0)	14% (4)	0% (0)	14	0% (0)	21% (3)	0% (0)
**0.5**	10	0% (0)	0% (0)	0% (0)	24	0% (0)	4% (1)	0% (0)	15	0% (0)	0% (0)	0% (0)
**1**	17	0% (0)	0% (0)	12% (2)	32	6% (2)	13% (4)	3% (1)	15	0% (0)	0% (0)	13% (2)
**10**	8	0% (0)	0% (0)	13% (1)	31	0% (0)	6% (2)	0% (0)	18	6% (1)	0% (0)	6% (1)
**100**	12	8% (1)	8% (1)	8% (1)	19	0% (0)	0% (0)	0% (0)	11	0% (0)	9% (1)	0% (0)
**1000**	11	0% (0)	0% (0)	0% (0)	24	0% (0)	4% (1)	4% (1)	10	0% (0)	0% (0)	0% (0)
**10000**	14	0% (0)	0% (0)	0% (0)	29	0% (0)	3% (1)	3% (1)	14	7% (1)	0% (0)	7% (1)

Data are presented as percentage. The percentages were rounded off to the whole number. n represents the number of cells used in each experiment. The value in the parenthesis represents the number of QES-EAD arrhythmia occurred.

Table S12. Occurrence of VTs in HCMT-CMs. Three types of VTs were observed in HCMT-CMs.

nM	Baseline	Adrenaline	Washout
**0.1** (n=29)	0	1/29<br>1:NRVT	0
**0.5** (n=24)	1/24<br>1: NSVT	3/24<br>1: NSVT<br>2: SVT	3/24<br>2: NSVT<br>1: SVT
**1** (n=32)	0	3/32<br>3: NSVT	2/32<br>2: NSVT
**10** (n=31)	0	1/31<br>1:SVT	3/31<br>2: NSVT<br>1: NRVT
**100** (n=19)	0	0	1/19<br>1: NSVT
**1000** (n=24)	0	0	1/24<br>1:NSVT
**10000** (n=29)	0	0	0

n represents the number of cells used in each experiment.

SVT: Sustained VT

NSVT: Non-sustained VT

NRVT: Non-recovered VT

Disease Models & Mechanisms • Supplementary information

Table S13. Effect of various concentrations of adrenaline in variabilities in WT-CMs.

Adrenaline	Δ%SD1	Δ%SD2	Δ%SDRR	Δ%SDSD	Δ%STV-APD90	Δ%STV-APD50
**0.1 nM** (n=11)	10.84±12.52	-3.16±7.40	1.17±8.26	10.84±12.52	-6.50±8.74	3.70±7.40
**0.5 nM** (n=8)	-1.83±10.08	-12.22±7.29*	-9.69±6.80	-1.83±10.08	-9.00±6.42	-4.00±5.14
**1 nM** (n=14)	-2.11±11.77	2.64±11.90	-0.81±9.44	-2.11±11.77	-7.53±4.47	-7.52±5.14
**10 nM** (n=13)	-27.39±5.34**	-28.84±7.95**	-30.88±6.52**	-27.39±5.34**	-23.81±6.36**	-23.28±5.87**
**100 nM** (n=11)	-36.09±7.90	-35.21±8.19*	-35.76±7.70*	-36.09±7.90	-23.15±6.27**	-23.77±6.12**
**1uM** (n=7)	-42.46±8.81*	-41.45±12.48	-44.36±11.27	-42.46±8.81*	-25.85±5.74**	-25.16±6.11**
**10 uM** (n=10)	-49.66±8.02***	-52.45±9.70*	-55.91±8.02**	-55.65±5.96***	-35.99±8.20**	-36.36±8.47**

The percentage change was calculated with respect to baseline (pre-adrenaline) value. Data are presented as mean±SEM. * $P < 0.05$, ** $P < 0.005$ and *** $P < 0.0001$ (paired *t*-test, pre adrenaline vs. post adrenaline) n represent the number of cells used in each experiment. The '–' sign means that the value had decreased from its corresponding pre-drug administration.

Disease Models & Mechanisms • Supplementary information

Table S14. Effect of various concentrations of adrenaline in variabilities in HCMT-CMs.

Adrenaline	Δ%SD1	Δ%SD2	Δ%SDRR	Δ%SDSD	Δ%STV-APD90	Δ%STV-APD50
**0.1 nM** (n=17)	22.14±9.90	23.96±12.36	22.93±11.21	22.14±9.90	2.59±6.98	9.32±5.24
**0.5 nM** (n=22)	-8.09±9.24	-9.85±9.18	-10.39±8.85	-8.09±9.24	-12.00±6.14	-13.46±6.21
**1 nM** (n=16)	-16.48±4.79**	-18.58±6.92*	-18.47±5.75**	-16.48±4.79**	-3.44±3.92	-4.04±4.05
**10 nM** (n=22)	-14.43±9.58	-17.09±12.22	-16.64±11.32*	-14.43±9.58*	-5.64±3.99*	-8.27±3.59*
**100 nM** (n=13)	-33.30±4.93*	-2.75±12.97	-14.68±8.84	-33.30±4.93	-27.50±5.36	-26.07±5.18
**1uM** (n=20)	-33.77±5.09***	-41.07±6.08***	-39.46±5.18***	-33.77±5.09***	-23.89±4.44**	-24.22±4.22**
**10 uM** (n=21)	-37.08±6.57***	-36.69±6.31***	-37.50±6.26***	-37.08±6.57***	-19.58±5.22*	-22.18±5.68*

The percentage change was calculated with respect to baseline (pre-adrenaline) value. Data are represented as mean±SEM. * $P < 0.05$, ** $P < 0.005$ and *** $P < 0.0001$ (paired *t*-test, pre adrenaline vs. post adrenaline) n represents the number of cells used in each experiment. The '–' sign means that the value had decreased from its corresponding pre-drug administration

Disease Models & Mechanisms • Supplementary information

Table S15. Effect of various concentration of adrenaline in variabilities in HCMM-CMs.

Adrenaline	Δ%SD1	Δ%SD2	Δ%SDRR	Δ%SDSD	Δ%STV-APD90	Δ%STV-APD50
**0.1 nM** (n=11)	30.70±15.23	7.27±11.57	12.85±11.54	30.70±15.23	-1.74±5.51	0.21±5.72
**0.5 nM** (n=13)	-6.46±11.21	0.92±10.58	-1.63±10.32	-6.46±11.21	-10.38±4.61	-10.08±5.17
**1 nM** (n=8)	-10.05±14.04	-17.34±18.51	-16.45±17.03	-10.05±14.04	-23.33±6.20	-23.03±5.80
**10 nM** (n=12)	-30.51±9.79*	-31.36±10.02	-32.28±8.75	-30.51±9.79*	-28.19±6.86	-27.17±6.98
**100 nM** (n=8)	-35.90±7.13*	-25.22±8.13*	-29.75±7.18*	-35.90±7.13*	-21.86±6.63*	-23.17±6.55*
**1uM** (n=7)	-42.16±11.75	-47.83±9.49*	-47.75±9.09*	-42.16±11.75	-20.92±8.87	-20.75±8.65
**10 uM** (n=9)	-33.78±5.81*	-40.39±5.41*	-37.66±5.38*	-33.78±5.81*	-11.58±8.15	-12.42±7.92

The percentage change was calculated with respect to baseline (pre-adrenaline) value. Data are presented as mean±SEM. * $P < 0.05$, (paired *t*-test, pre adrenaline vs. post adrenaline) n represents the number of cells used in each experiment. The '–' sign means that the value had decreased from its corresponding pre drug administration

Disease Models & Mechanisms • Supplementary information

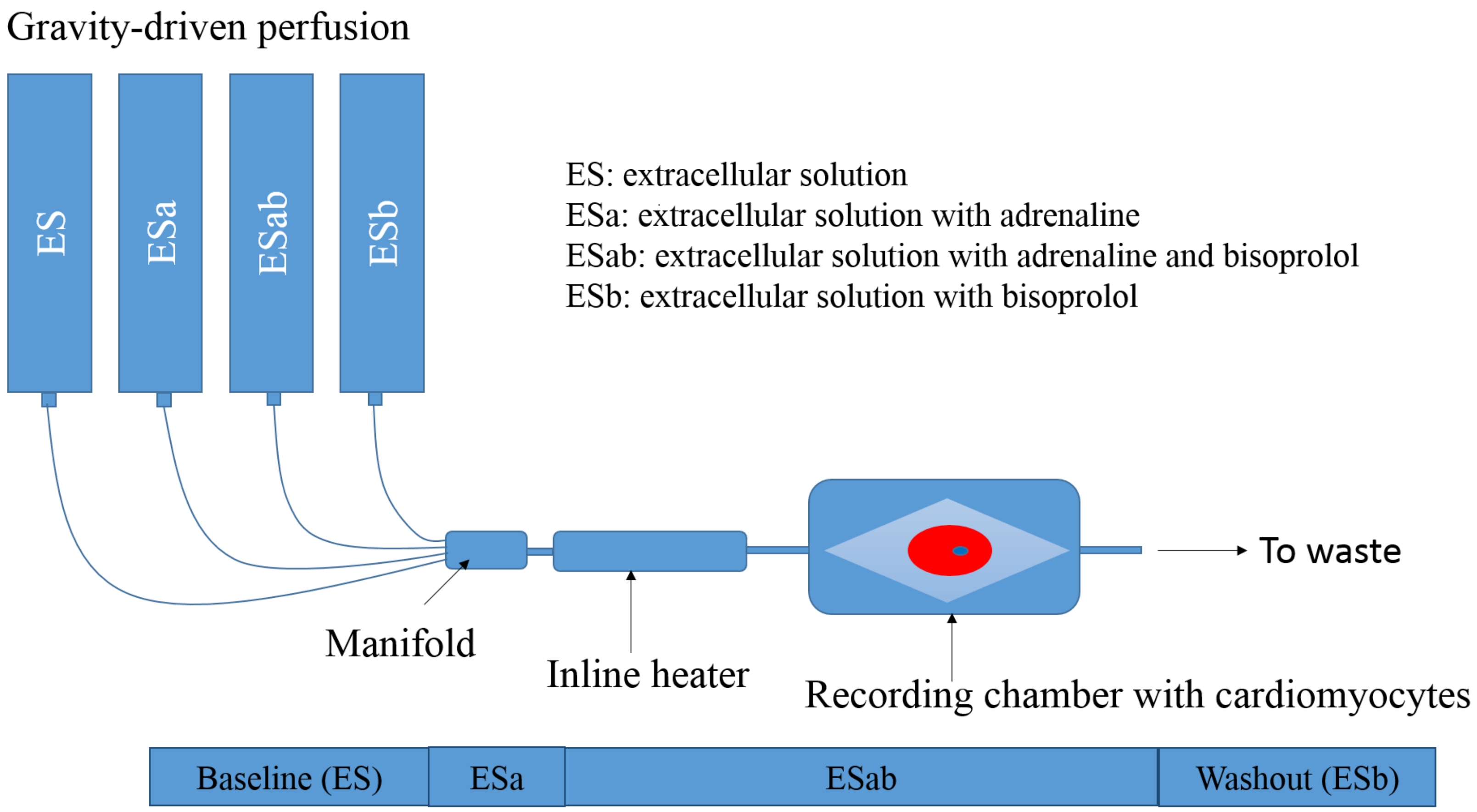

Figure S9. Experimental protocol for adrenaline and bisoprolol testing. After the stable recording, adrenaline alone (ESa) was passed for 1-2 minute after which extracellular solution containing both adrenaline and bisoprolol (ESab) was passed. Subsequently, extracellular solution only with bisoprolol (ESb) was used. To study the efficacy of bisoprolol, APs were chosen from recording after minimum of 3-minute perfusion of solution with adrenaline and bisoprolol (ESab). Furthermore, to study the potency of bisoprolol in washing out condition of adrenlaine, maximum up to 300ms immediately after the adrenaline being washout were selected for analysis.

Disease Models & Mechanisms • Supplementary information
